# Cost-effectiveness analysis of guideline-based optimal care for venous leg ulcers in Australia

**DOI:** 10.1186/s12913-018-3234-3

**Published:** 2018-06-07

**Authors:** Qinglu Cheng, Michelle Gibb, Nicholas Graves, Kathleen Finlayson, Rosana E. Pacella

**Affiliations:** 1 Institute of Health and Biomedical Innovation, Queensland University of Technology, Brisbane, Queensland Australia; 2 Wound Specialist Services, Brisbane, Queensland Australia; 3 Wound Management Innovation Cooperative Research Centre, Brisbane, Queensland Australia

**Keywords:** Cost-effectiveness, Markov model, Venous leg ulcer, Guideline-based care, Wound management

## Abstract

**Background:**

Venous leg ulcers (VLUs) are expensive to treat and impair quality of life of affected individuals. Although improved healing and reduced recurrence rates have been observed following the introduction of evidence-based guidelines, a significant evidence-practice gap exists. Compression is the recommended first-line therapy for treatment of VLUs but unlike many other developed countries, the Australian health system does not subsidise compression therapy. The objective of this study is to estimate the cost-effectiveness of guideline-based care for VLUs that includes public sector reimbursement for compression therapy for affected individuals in Australia.

**Methods:**

A Markov model was designed to simulate the progression of VLU for patients receiving guideline-based optimal prevention and treatment, with reimbursement for compression therapy, and then compared to usual care in each State and Territory in Australia. Model inputs were derived from published literature, expert opinion, and government documents. The primary outcomes were changes to costs and health outcomes from a decision to implement guideline-based optimal care compared with the continuation of usual care. Sensitivity analyses were performed to test the robustness of model results.

**Results:**

Guideline-based optimal care incurred lower total costs and improved quality of life of patients in all States and Territories in Australia regardless of the health service provider. We estimated that providing compression therapy products to affected individuals would cost the health system an additional AUD 270 million over 5 years but would result in cost savings of about AUD 1.4 billion to the health system over the same period. An evaluation of unfavourable values for key model parameters revealed a wide margin of confidence to support the findings.

**Conclusions:**

This study shows that guideline-based optimal care would be a cost-effective and cost-saving strategy to manage VLUs in Australia. Results from this study support wider adoption of guideline-based care for VLUs and the reimbursement of compression therapy. Other countries that face similar issues may benefit from investing in guideline-based wound care.

**Electronic supplementary material:**

The online version of this article (10.1186/s12913-018-3234-3) contains supplementary material, which is available to authorized users.

## Background

The prevalence of venous leg ulcers (VLUs) increases with age and although there are no recent or nationwide studies in Australia, prevalence is estimated at 0.33% in people aged over 60 years based on a study in Western Australia from the early 1990s [1]. However, this common and recurrent problem has likely increased in recent years with population ageing. VLUs are expensive to treat and require extensive health care resources such as frequent dressing changes by health professionals. The average cost of VLU treatment per patient in 2012–13 in Australia was estimated to be AUD 8106 [2]. Patients with VLUs also experience impaired quality of life (QoL) as a result of significant pain, restricted mobility and limited ability to work [3].

In Australia, the Australian and New Zealand Clinical Practice Guideline for Prevention and Management of Venous Leg Ulcers presents a comprehensive review of the assessment, diagnosis, management and prevention of VLUs based on best available evidence [4]. Even though improved healing and reduced recurrence rates have been observed following the introduction of evidence-based guidelines [5], a significant evidence-practice gap exists. Although the guideline recommends the use of compression therapy for both treatment and prevention of VLUs, studies found that 40–60% of VLUs in Australia did not receive adequate compression therapy [6, 7]. This gap is attributable to lack of information, skills and reimbursement [8, 9]. Medicare, Australia’s universal health insurance scheme, funded by the Australian Government, reimburses health care provided by general practitioners (GPs), medical specialists and nurse practitioners outside hospital as per the Medicare Benefits Schedule (MBS) [10]. Unlike many other developed countries, in Australia, compression therapy is not subsidised through MBS or Pharmaceutical Benefits Scheme (PBS) [11] which lists the medical services and medicines subsidized by the Australian Government. Only veterans who have served in the Australian Defence Force covered by the Repatriation Schedule of Pharmaceutical Benefits (RPBS) receive subsidies for compression bandages. The out-of-pocket costs for VLU patients over 60 years old have been estimated at AUD 27.5 million per year in total [2].

There is also little evidence on whether guideline-based care for VLU is cost-effective and represents good value for money in Australia. One study in Australia recently conducted an economic evaluation of compression therapy for VLUs [2]. The study modelled treatment pathways for VLUs and determined the expected cost of treatment per patient and per wound for compression and non-compression therapies. Results from the model demonstrated that patients undergoing compression therapy would incur fewer costs than those treated without compression therapy. Unfortunately, the study did not include other important recommendations of evidence-based care. Recurrence was not taken into account in the model, hence prevention of VLUs was not considered, and it was assumed that patients treated with compression therapy would not experience complications that required hospitalisation. The total costs of managing VLUs may therefore have been underestimated in the compression group.

In response to the need for evidence on cost-effectiveness, we conducted a comprehensive economic evaluation of guideline-based care for VLUs by State and Territory in Australia drawing on multiple data sources including data from previous economic evaluations. The aim was to estimate the cost-effectiveness of guideline-based optimal prevention and treatment of VLUs in Australia that includes reimbursement for compression therapy for patients with an active ulcer and patients with a healed ulcer and history of VLU to prevent recurrence.

## Methods

This economic evaluation conforms to the guidelines outlined in the Consolidated Health Economic Evaluation Reporting Standards (CHEERS) statement [12], with regard to reporting of methods and results (Additional file 1: Table S11).

### Description of two competing systems of care for VLUs

#### Optimal care

Optimal care refers to a situation where all individuals with VLUs receive guideline-based care with appropriate MBS and PBS reimbursement linked to compression therapy provided by accredited wound care providers assuming 100% adherence. However, it is possible that even with economic incentives and encouragement, some patients will never consistently wear compression therapy as required and others will be adherent without these incentives. Clinical practice under optimal care follows the Australian and New Zealand Clinical Practice Guideline which has a flow chart of evidence-based recommendations including compression therapy for the prevention and management of VLUs [4]. The key elements of optimal care are summarised in Additional file 1: Table S1.

#### Optimal wound care service delivery

We assumed that optimal wound care services could be delivered through either: Option 1) specialist wound clinics led by nurse practitioners with wound expertise together with a team of allied health professionals and specialists; or Option 2) GPs, community nursing services and outpatient clinics by clinicians trained in evidence-based wound care.

#### Usual care

Usual care refers to a situation where individuals do not receive all the components of guideline-based care listed under optimal care. Since a small proportion of Australians are currently receiving compression therapy we tried to model a situation where usual care would also include a proportion of patients receiving compression therapy but with partial adherence and no reimbursement hence incurring substantial out-of-pocket costs. Since different funding, reimbursement arrangements and cost structures apply to different health care providers in different States and Territories in Australia, patient out-of-pocket payments for wound care also vary depending on these arrangements and structures (Additional file 1: Table S3).

### Markov model simulation

**Fig. 1:**
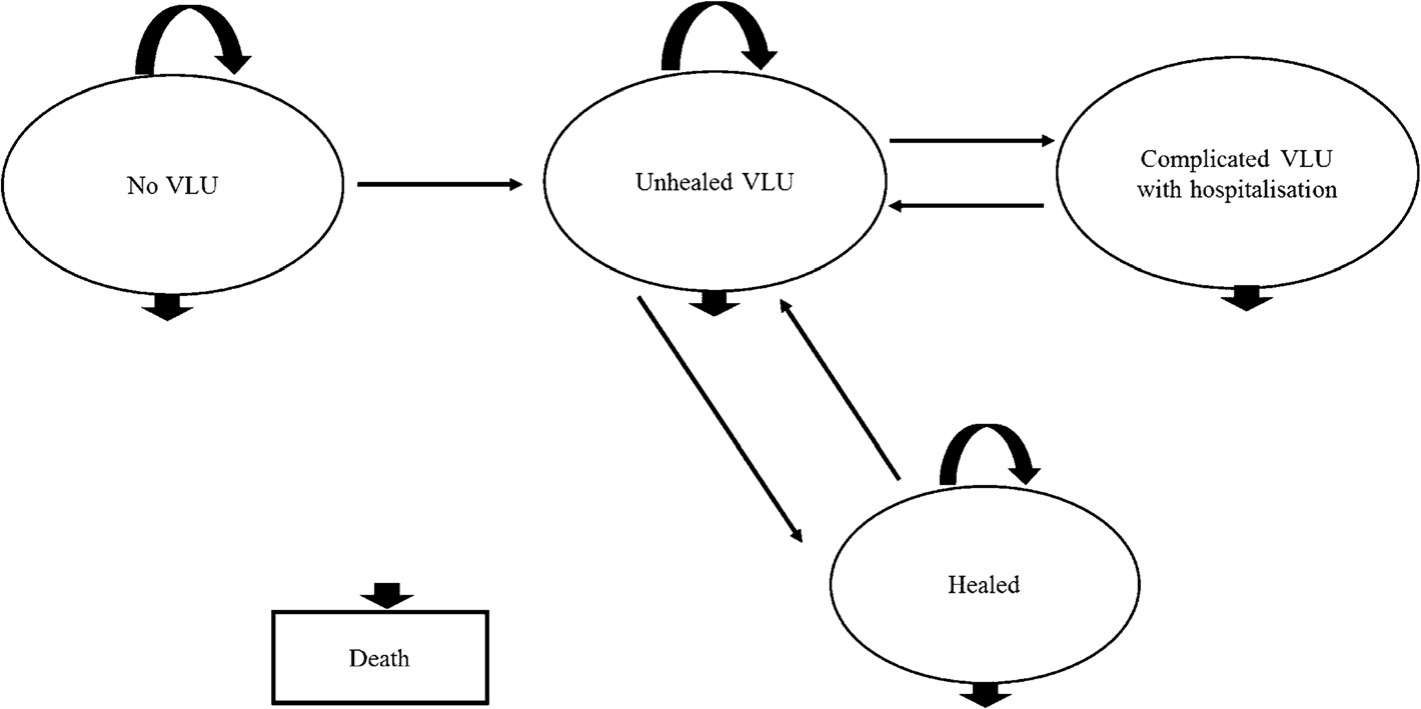
Markov model structure for assessing the cost-effectiveness of guideline-based optimal care for VLUs

Building on our previous model [13], a decision-analytic Markov model was developed to assess the cost-effectiveness of guideline-based optimal care for VLUs in each of the eight States and Territories in Australia (New South Wales (NSW), Victoria (VIC), Queensland (QLD), South Australia (SA), Western Australia (WA), Northern Territory (NT), Tasmania (TAS), and Australian Capital Territory (ACT)). In this study, five mutually exclusive health states were identified for the model: ‘No VLU’, ‘Unhealed VLU’, ‘Healed’, ‘Complicated VLU with hospitalisation’ and ‘Death’ and individuals can transit between these states (Fig. 1). One advantage of Markov models is that recurrent events over time can be modelled [14]. Thus, in our model, individuals could experience more than one VLU or hospitalisation.

The model ran on fortnightly cycles for a total of 130 cycles or 5 years. A time horizon of 5 years was chosen to incorporate long-term recurrence. Since VLU mainly affects the elderly population, the target population entering the model would be the general population aged 60 years and over in each State and Territory. At cycle 0, 0.33% of the population would be distributed to ‘Unhealed VLU’ state to represent current prevalence of VLUs. The remaining population would enter the model from ‘No VLU’ state referring to no previous or current VLU in this population but at risk of developing VLUs. After one cycle (i.e. a fortnight), people in ‘No VLU’ state could develop an ulcer and enter ‘Unhealed VLU’ state, remain in ‘No VLU’ state or die from all causes. Meanwhile, those with VLUs could either become ‘Healed’, develop ‘Complicated VLU with hospitalisation’, remain in the ‘Unhealed VLU’ state or die of all causes. Those patients that moved to the ‘Healed’ state would be at risk of recurrence, and likely to develop another VLU and return to ‘Unhealed VLU’ state in the next cycle. For those hospitalised, we assumed that all would be discharged from hospital after one cycle (a fortnight) or die of all causes. To take into account the aging population in Australia and obtain a more real-world estimate, the general population that gradually turns 60 was added to the ‘No VLU’ state cycle by cycle as new at-risk population. The average population growth for population aged 60 years and over was calculated using Australian Bureau of Statistics data [15] (as explained in more detail in the Additional file 1). The growth rate was not applied to cohorts in other health states.

The transitions between states depended on transition probabilities (i.e. incidence, probability of healing, probability of recurrence, probability of hospitalisation, probability of death). Costs and health outcomes (expressed in quality-adjusted life years (QALYs)) associated with each health state per cycle were incorporated into the model and were accumulated over 130 cycles. Future costs and QALYs were discounted at 5% in baseline analyses in line with Australian government guidelines [16, 17]. All analyses were conducted using Microsoft Excel 2010.

### Data used for the model

#### Transition probabilities

A comprehensive review of literature was conducted to source transition probabilities for the model. The incidence of VLU in older persons has not been well established in Australia. We used an annual incidence rate of 1.2 cases per 100 person-years from a UK study where the incidence of VLUs among people aged 65 years and older was reported [18] and applied this to the Australian population as summarised in Table 1.

**Table 1:** Markov model inputs

Variables	Value	References
Transition probabilities^a^		
*Usual care*		
Probability of healing (3-month)	0.2281	[19]
Probability of recurrence (annual)	0.5574	[20]
Probability of hospitalisation (5-year)	0.24	[22]
*Optimal care*		
Probability of healing (3-month)	0.5872	[19]
Probability of recurrence (annual)	0.2222	[5]
Probability of hospitalisation (annual)	0.0116	[21]
VLU Incidence rate (annual)	0.0121	[18]
All-cause mortality rate (annual)	0.0282	Calculated from ABS data [15]
Proportion of unhealed ulcers with infection		
*Usual care*	10%	[24]
*Optimal care*	5%	[24]
Cost items^b^		
GP	AUD 71.70	MBS item 36
Nurse practitioner	AUD 58.55	MBS item 82215
Vascular surgeon	AUD 85.55	MBS item 104
Community nurse hourly wage rate	AUD 24.74–37.05	[31–38]
Ankle-brachial pressure index (ABPI) assessment	AUD 63.74	MBS item 11610
Pathology test	AUD 33.75	MBS item 69306
Compression bandage	AUD 51.5	Retail price
Compression stocking	AUD 97.75	Retail price
Complicated VLU with hospitalisation	AUD 18,331.60	[23]
Quality of life utility score		
Healed VLU	0.75	[25]
Unhealed VLU	0.64	[25]
Complicated VLU with hospitalisation	0.54	calculated

^a^Yearly or monthly probabilities were transformed to fortnightly probabilities by the formula: tp = 1 – (1 - tp_t_)^1/t^ [14]

^b^Costs for health states by State and Territories in Australia are presented in Additional file 1 Table S4

The probability of ulcer healing in the optimal care group and usual care group were obtained from Harrison et al. [19]. In the Harrison et al. [19] study in Canada, 3-month healing rates for VLUs more than doubled between the year before (22.8% [13/57]) and after (58.7% [64/109]) implementation of an evidence-based service. In the same study, use of compression for VLUs increased from 43.9% (25/57) in the old model of care to 85.3% (93/109) after implementation of the new model. In a small study in Queensland, Edwards et al. [5] reported that 84% of patients with a VLU were treated with compression therapy following admission to a specialist wound clinic while only 6.3% (2 of 32) of patients with a venous leg ulcer were receiving compression on admission to the clinic and a total of 11% had been treated with compression therapy at any time in the previous 12 months prior to admission. Healing was significantly associated with implementation of compression therapy with 63% (*n* = 20) of the participants with venous leg ulcers healed by 3 months, although this study did not report healing rates prior to admission to the specialist wound clinic. Since Edwards et al. [5] reported similar healing rates with implementation of evidence-based services as Harrison et al. [19], and the two studies reported similar use of compression therapy after implementation of evidence-based care, healing rates from the Harrison et al. [19] study were used in this analysis for optimal care and usual care as we were assuming that about 50% of patients in usual care group receive compression therapy.

Recurrence rates from Edwards et al. [5] and Finlayson et al. [20] were used to inform probability of ulcer recurrence in the optimal care group and usual care group, respectively. In the absence of local data, probabilities of hospitalisation due to VLU complications in the optimal care group and usual care group were derived from studies by Barwell et al. [21] and Walker et al. [22], respectively. All admissions to hospital were assumed to occur in public hospitals as Independent Hospital Pricing Authority (IHPA) mainly collects data from public hospitals [23]. For individuals that transited to or remained in ‘Unhealed’ state, we assumed that 5% of them would have infected VLU if they received optimal care and 10% of them would have an infection if they received usual care [24]. Since there is insufficient evidence for an increased risk of mortality with VLU, all-cause mortality rates for people aged 60 years and over were calculated using aged-specific deaths and age-specific population by State and Territory from the Australian Bureau of Statistics dataset [15]. Yearly and monthly probabilities were then transformed to fortnightly probabilities [14]. Because the probabilities of moving between states in each cycle must sum up to 1, the probability of staying in a state was one minus the sum of the probabilities of leaving the state.

#### Resources use and costs

Costs associated with health states and transitions in the Markov model were measured in 2015 Australian dollars. This study was conducted from a partial societal perspective. Patients’ out-of-pocket costs were calculated. But costs of lost productivity were not considered in this study as we expected that most patients aged 60 years and over would be retired. We also did not include the cost of education and training of health professionals in evidence-based practice in optimal care Options 1 and 2. Resource use in usual care group and optimal care group is summarised in Table 2 with details and assumptions presented in the Additional file 1.

**Table 2:** Summary of resource use in usual care group and optimal care group

	Usual care	Optimal care (Option 1)	Optimal care (Option 2)
Healed VLU	No additional care	• Clinic assessment by nurse practitioner every 3 months • Compression stocking every 3 months	• Clinic assessment by GP every 3 months • Compression stocking every 3 months
Unhealed VLU	• One-off assessment by GP • Clinic visits to GP or community nurse or outpatient clinic twice a week • Dressings change twice a week • High compression therapy prescribed to 50% of patients If infected • Pathology test every week • systemic antibiotics	• One-off ABPI or vascular assessment and assessment by nurse practitioner and vascular surgeon • Clinic visits to nurse practitioner once a week • Dressings change every week • High compression therapy prescribed to everyone If infected • One-off pathology test • Debridement once a week • Systemic antibiotics	• One-off ABPI and GP assessment • Clinic visits to GP or community nurse or outpatient clinic once a week • Dressings change every week • High compression therapy prescribed to everyone If infected • One-off pathology test • Debridement once a week • Systemic antibiotics

#### QALYs

QoL utility scores for ‘Unhealed VLU’ and ‘Healed’ state were sourced from lglesias et al. [25]. As we could not identify information on the QoL of patients hospitalised due to VLUs, we assumed a decrement of 0.1 utility score for ‘Complicated VLU with hospitalisation’ state as done by one National Institute for Health and Care Excellence guideline [26]. QALYs were calculated by multiplying the utility score by the amount of time spent in that health state.

### Model outputs

The primary outcome measures analysed in the model were the expected total costs and QALYs associated with optimal care versus usual care in each State and Territory over 5 years. Total costs were separated into costs covered by the health system and out-of-pocket costs. The Australian government and State and Territory government share of the health system costs was estimated. Expected costs of compression therapy were calculated for both groups. Assuming that public hospitals are funded by both State and Territory governments (60%) and the Australian government (40%) [27] and MBS and PBS costs as well as cost of products for compression therapy under optimal care are covered by the Australian government, we also calculated the cost savings to State and Territory governments and the Australian government respectively.

### Deterministic sensitivity analysis

There is always uncertainty in the estimation of model inputs of interest such as healing rate and hospitalisation rate. One-way sensitivity analysis was therefore conducted where model inputs were varied between a low value and high value using a uniform distribution and corresponding model outputs were recorded. The model input that had the greatest impact on the outcomes was evaluated further in a scenario analysis.

### Probabilistic sensitivity analysis

Uncertainty around model inputs was quantified using probabilistic sensitivity analysis (PSA) to give the decision maker insight into the probability that a change to practice will be cost-effective. Statistical distributions were used to describe variability in the model inputs. Transition probabilities and quality of life utility scores were assigned beta distributions. Gamma distributions were used for cost parameters to reflect the skew typically found in cost data. 10,000 Monte Carlo simulations were performed for PSA. In each simulation, the model parameters took random values from the fitted distribution, and the economic outcomes of change to costs (ΔC) and change to health benefits or QALYs (ΔE) were calculated. Net monetary benefit (NMB) was then calculated by NMB = ΔE* λ - ΔC where λ is the decision maker’s maximum willingness-to-pay threshold for an additional unit of health benefit gain. A value of $64,000 per QALY was used for willingness-to-pay in this study, in line with research in an Australian setting [28]. NMB greater than 0 indicates that optimal care is cost-effective in this simulation. The results of 10,000 simulations were presented using a cost-effectiveness plane. The probability that optimal care was cost-effective at certain willingness-to-pay thresholds was derived by counting the number of times out of 10,000 that NMB was greater than 0 and summarised in cost-effectiveness acceptability curves.

## Results

### Baseline analysis

By the end of 2020, we estimated that there would be over 300,000 people affected by VLU in Australia which would include new and existing cases over that period as well as individuals with a healed ulcer that have a history of VLU. This should not be expressed as the number of existing cases at the end of 5 years as it does not take into account mortality or healed cases over that 5-year period. Estimated number of people affected by VLU and hospitalisation in each State and Territory is presented in Additional file 1: Table S5. Number of hospitalisations avoided through optimal care over 5 years was estimated at 21,677 nationwide.

In optimal care service delivery Option 1 where optimal care was provided by specialist nurse practitioners, the expected total cost for all affected persons over 5 years was over AUD 1.2 billion under optimal care and over AUD 2.8 billion under usual care (Table 3). In addition to lowered costs, the society would witness improved quality of life among patients managed by optimal care. Total costs were broken down to costs borne by the health system and out-of-pocket costs by patients. Following guideline-based optimal care would always be a cost-saving strategy for the health system. Although there are currently no specific MBS item numbers for compression therapy, under usual care, the health system is already paying an estimated 270 million for compression products indirectly. Under optimal care, funding consumables for compression therapy for treatment of patients with existing VLUs and prevention to patients with a history of VLUs would cost the Australian government an additional 270 million over 5 years. The cost savings to the Australian government through reduced health service utilisation as a result of faster healing of wounds, ulcers avoided and hospitalisations avoided would be about AUD1.2 billion (85% of cost-savings to the health system) and to state and territory governments about AUD 200 million over 5 years through reductions in hospitalisations due to complications.

**Table 3:** Baseline outcomes for all affected persons over 5 years in Australia (optimal care versus usual care)^c^

	Option 1	Option 2
Total costs in usual care group	$2,831,006,652	$2,831,006,652
Total costs in optimal care group	$1,200,427,937	$1,126,566,791
Total incremental costs	-$1,630,578,715	-$1,704,439,861
Health system costs in usual care group	$2,516,869,902	$2,516,869,902
Health system costs in optimal care group	$1,128,856,145	$1,093,241,400
Incremental costs	-$1,388,013,757	-$1,423,628,502
Costs by Australian government in usual care group	$2,298,900,749	$2,298,900,749
Costs by Australian government in optimal care group	$1,114,692,967	$1,079,078,223
Incremental costs	-$1,184,207,781	-$1,219,822,526
Costs by State and Territory government in usual care group	$217,969,153	$217,969,153
Costs by State and Territory government in optimal care group	$14,163,177	$14,163,177
Incremental costs	-$203,805,976	-$203,805,976
Out-of-pocket costs in usual care group	$314,136,750	$314,136,750
Out-of-pocket costs in optimal care group	$71,571,792	$33,325,391
Incremental costs	-$242,564,957	-$280,811,359
Total costs of compression products in usual care group^a^	$487,379,273	$487,379,273
Total costs of compression products in optimal care group^b^	$539,765,074	$539,765,074
Incremental costs	$52,385,801	$52,385,801
Costs of compression products in usual care group indirectly covered by health system	$271,197,256	$271,197,256
Total costs of compression products in optimal care group covered by health system	$539,765,074	$539,765,074
Incremental costs	$268,567,818	$268,567,818
Costs of other dressings in usual care group	$37,875,018	$37,875,018
Costs of other dressings in optimal care group	$68,758,106	$68,758,106
Incremental costs	$30,883,088	$30,883,088
Total QALYs in usual care group	476,090	476,090
Total QALYs in optimal care group	504,431	504,431
Incremental QALYs	28,341	28,341

^a^Total costs of compression products in usual care group were covered by out-of-pocket expenditure and government’s funding to health professionals

^b^Costs of compression products in optimal care group were covered by Australian government

^c^Costs were measured in 2015 Australian dollars. Baseline outcomes by State and Territory are summarised in Additional file 1 Tables S6 and S7. Costs per person and QALY per person could not be calculated by dividing the total by the number of affected persons as not all affected persons received treatment and prevention for the entire 5 years in this model. By the end of 2020, patients who developed VLUs in 2016 would incur more costs associated with treatment and prevention than those who developed VLUs in 2017. Expected costs and QALYs per person over 5 years if the person received treatment and prevention for 5 years are summarised in Additional file 1: Tables S8 and S9

The optimal care service delivery Option 2 (guideline-based wound care delivered by trained GP, community nurses, outpatient clinics) analysis showed similar results to Option 1. Guideline-based optimal care is a cost-effective and cost-saving strategy as it costs less than usual care and generated more QALYs even after factoring in the additional costs of compression therapy under guideline-based optimal care. The costs borne by the health system under second option of optimal care service delivery were slightly lower than under Option 1 (specialist wound clinic with nurse practitioner) since a proportion of care under Option 2 (GP, community nursing, outpatient clinics) was provided by community nurses at lower costs. Patients also incurred lower out-of-pocket costs when receiving optimal care at GP clinic, community or outpatient clinics compared with specialist clinics as patients would receive subsidies for other consumables used according to Additional file 1: Table S3. Nevertheless, cost savings to health system are similar at around AUD 1.4 billion (AUD 1.2 billion to Australian government and AUD 200 million to State and Territory) with optimal care for VLU.

### Deterministic sensitivity analysis

**Fig. 2:**
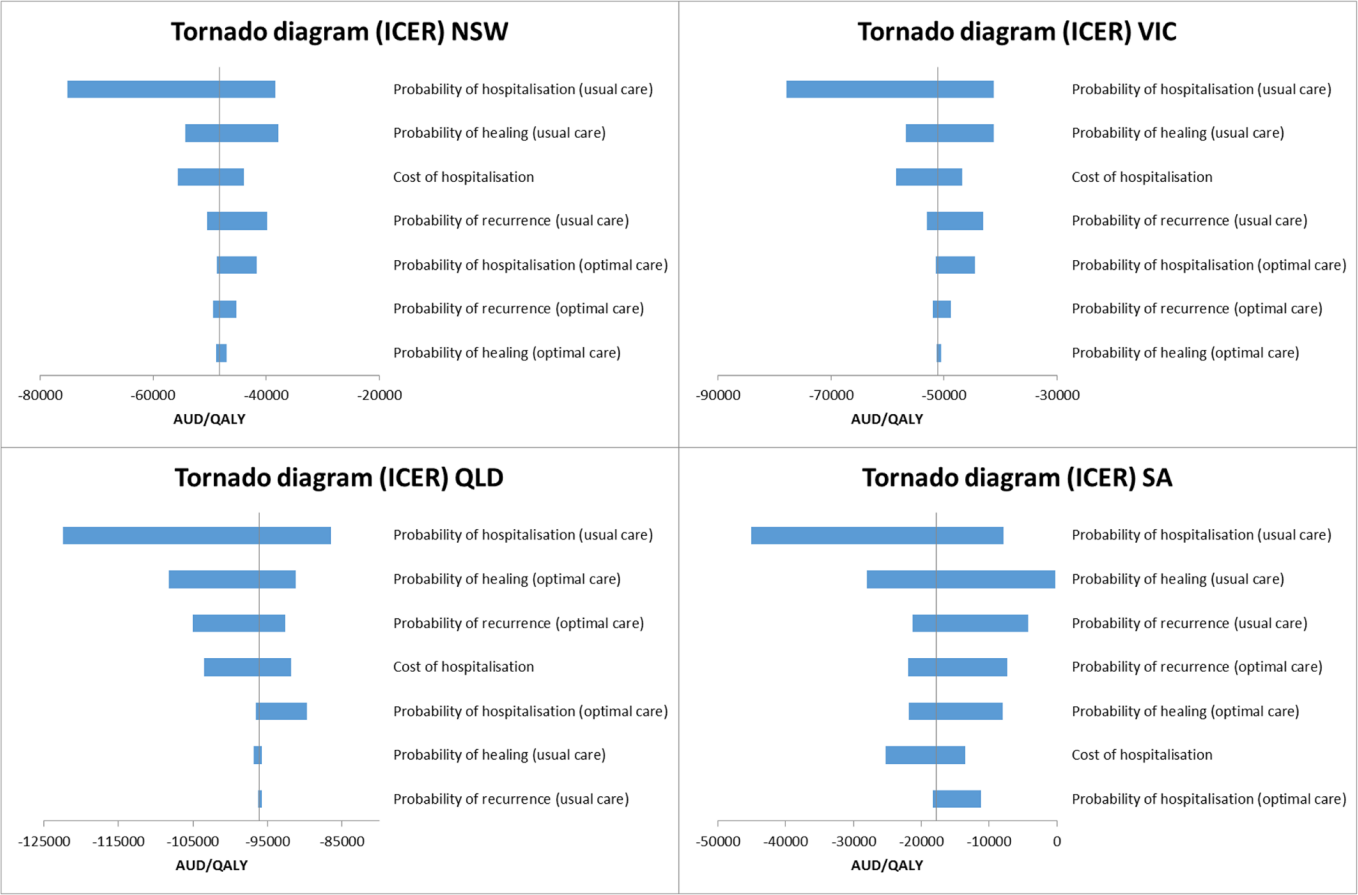
Tornado diagrams for NSW, VIC, QLD, SA (optimal care service delivery Option 1)

**Fig. 3:**
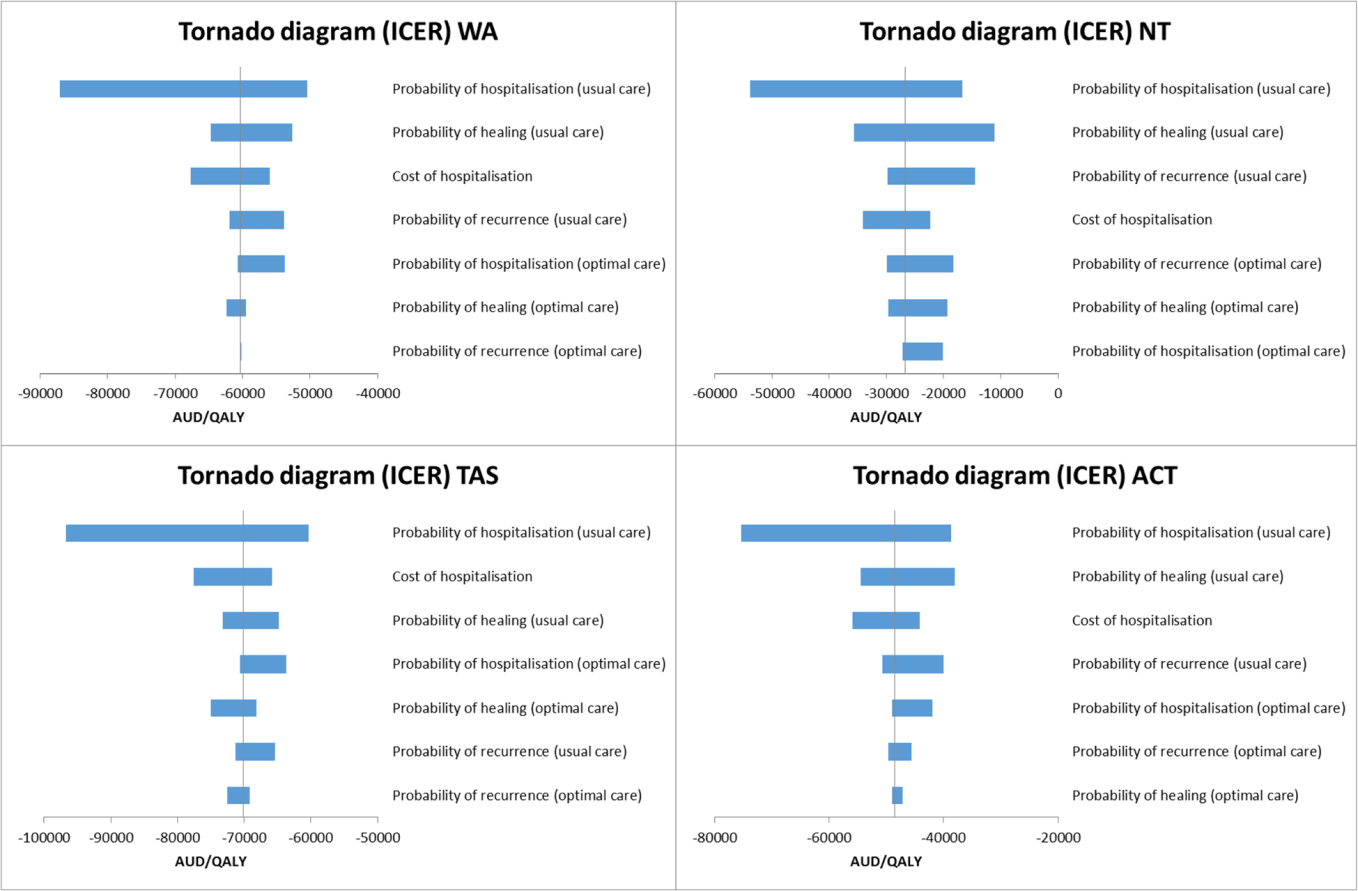
Tornado diagrams for WA, NT, TAS, ACT (optimal care service delivery Option 1)

Results of one-way sensitivity analyses under Option 1 as an example are summarised using tornado diagrams (Figs. 2 and 3) where each bar represents one one-way sensitivity analysis and the width of the bar shows the extent of impact on model results. The vertical line denotes ICER from baseline analysis. The tornado diagram indicates that varying values of hospitalisation rate in usual care group would greatly change the amount of benefit gained whereas varying the probability of healing or recurrence to less favourable values has less impact.

Scenario analysis was then conducted to test how different values of hospitalisation rate (per annum) in usual care group influenced the costs and health outcomes of optimal care compared with usual care (Table 4). The lowest value tested was the same as hospitalisation rate in optimal care group (1.16%). The high value of probability of hospitalisation in the usual care group was calculated using the number of VLU hospitalisations divided by the VLU population aged 60 and over. The number of VLU hospitalisations was based on hospitalisations associated with leg ulcer complications such as cellulitis as per Australian Refined Diagnosis Related Groups (AR-DRG codes J12A and J12B) [23]. The results suggested that the higher the hospitalisation rate, the higher the cost savings and avoided hospitalisations, but the analysis shows cost savings even when using the lowest value of hospitalisation rate.

**Table 4:** Scenario analysis varying hospitalisation rate in usual care group (optimal care service delivery Option 1)^a^

Hospitalisation rate in usual care group (annual)	5.34% (Baseline)	1.16% (low)	12.36% (high)
Total costs in usual care group	$2,831,006,652	$2,543,990,422	$3,340,479,326
Total costs in optimal care group	$1,200,427,937	$1,200,427,937	$1,200,427,937
Total incremental costs	-$1,630,578,715	-$1,343,562,485	-$2,140,051,390
Total QALYs in usual care group	476,090	476,181	475,928
Total QALYs in optimal care group	504,431	504,431	504,431
Incremental QALYs	28,341	28,249	28,503
Hospitalisations avoided over 5 years	21,677	3461	54,013

^a^ Costs, QALYs and hospitalisations avoided were calculated for all individuals affected by VLU over 5 years in Australia

An additional scenario analysis was conducted for prevalence of VLUs. As there is no recent population study of the prevalence of VLUs in Australia, it was assumed that the prevalence could take values between 0.33 and 1.69% where the upper limit was the prevalence of VLU observed in elderly population in UK [18]. Table 5 summarises the costs and health outcomes of implementing optimal care compared with usual care in Australia under different values of prevalence. The results suggested that optimal care was always associated with lower costs and higher QALYs. The cost-savings would increase as prevalence of VLU increases.

**Table 5:** Scenario analysis varying prevalence of VLUs (optimal care service delivery Option 1)^a^

Prevalence of VLU among 60+	0.33% (Baseline)	0.50%	1%	1.69% [18]
Population 60+ affected with VLU at cycle 0	15,973	24,202	48,404	81,803
Total costs in usual care group	$2,831,006,652	$2,954,936,472	$3,319,435,945	$3,822,445,216
Total costs in optimal care group	$1,200,427,937	$1,246,526,209	$1,382,109,363	$1,569,214,116
Total incremental costs	-$1,630,578,715	-$1,708,410,263	-$1,937,326,581	-$2,253,231,100
Total QALYs in usual care group	476,090	499,250	567,367	661,369
Total QALYs in optimal care group	504,431	528,959	601,099	700,653
Incremental QALYs	28,341	29,709	33,732	39,284
Hospitalisations avoided	21,677	22,620	25,394	29,221

^a^Costs, QALYs and hospitalisations avoided were calculated for all individuals affected by VLU over 5 years in Australia

### Probabilistic sensitivity analysis

**Fig. 4:**
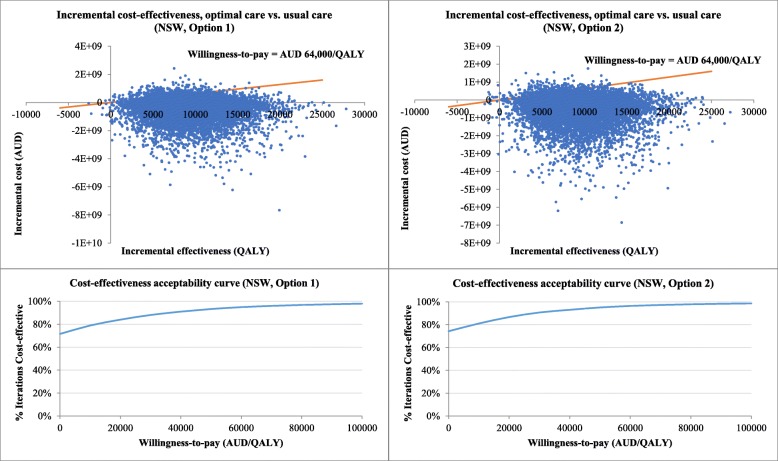
Probabilistic sensitivity analysis results in cost-effectiveness plane and cost-effectiveness acceptability curve (NSW as an example)

The 10,000 iterations of change to costs and health outcomes are displayed in Fig. 4 using NSW as an example. Each dot represents one iteration and most dots lie below the willingness-to-pay line, which means that optimal care has a high probability of being cost-effective when the willingness-to-pay is AUD 64,000/QALY. The probability of optimal care being cost-saving is about 70% when only dots in the southeast quadrant are counted. The probability that optimal care is cost-effective at different willingness-to-pay thresholds is shown in Fig. 4. The acceptability curve demonstrates that optimal care is associated with high probability (around 70–99%) of being cost-effective regardless of the willingness-to-pay value. The results for other States and Territories are presented in Additional file 1: Figures S1–S7.

## Discussion

While an economic evaluation of compression therapy has previously been conducted in Australia, this study breaks new ground in its attempt to measure the full health and economic impact of guideline-based care for VLUs by State and Territory in Australia. The model takes into account the growth in population aged 60+ years as well as new incident VLU cases that develop over time. It includes recurrent ulcers and complications resulting in hospitalisations in the model, to present a more comprehensive estimate of the cost savings in Australia with guideline-based optimal prevention and treatment for VLU. The study shows that over 5 years, optimal care will offset the costs of additional investment in compression therapy for treatment and prevention across all jurisdictions and save the health system about AUD 1.4 billion. The two key drivers of the estimated cost savings are reductions in healing time and rates of hospitalisation to treat complications. These improvements in quality of life for patients coupled with the reduced costs of guideline-based care for VLUs can be said to dominate as a prevention and treatment pathway for VLU. This study contributed further evidence to the literature on the cost-effectiveness of guideline-based wound management.

One strength of this study is that two options of delivering guideline-based care were considered in the analysis. GP and community nurses have been at the forefront of managing wounds in Australia [5], but evidence-based care that follows official guidelines is not widely practiced in primary care. For example, applying compression therapy to VLUs requires additional time and expertise from health professionals while the length of consultation in general practice is limited. Although we did not include this additional consultation time in our analysis, we modelled the situation where GP and community nurses are also capable of delivering optimal wound care. In most jurisdictions, there are few out-of-pocket costs for consumables in community nursing and outpatient clinics. Specialist wound clinics, on the other hand, provide evidence-based treatment and prevention but patients may be expected to pay additional costs for accessing specialist services and consumables. Results from this study indicated that both options could be cost-saving in the long-term as long as the health professionals are well-trained and equipped with knowledge and skills to manage VLUs and there is adequate reimbursement for clinician time and consumables through the health system. Improving education of patients and training of health professionals in wound management with adequate reimbursement of wound care products and services to increase uptake of evidence-based care remains a priority in Australia.

It is important to note patients’ out-of-pocket expenditure when optimal care is implemented. Overall we estimated that under usual care, VLU patients over 60 years of age would pay more than AUD300 million in out-of-pocket costs over 5 years. We estimated that the out-of-pocket costs could be reduced substantially under optimal care but the savings varied across jurisdictions. Patients in Queensland and Victoria had higher savings than other jurisdictions as a large proportion of consumable costs are paid by patients in these States, while there is little expected benefit in the ACT as consumable costs are already fully subsidised. The out-of-pocket costs under optimal care also depended on service delivery: for Option 1 (specialist wound clinics led by nurse practitioners) these costs (AUD72 million) are more than double that under Option 2 (AUD35 million) where care is provided by GPs, community nurses and outpatient clinics. Although we assumed that compression therapy consumables would be reimbursed by the health system under optimal care, patients still had to pay a small amount for medicines listed on the PBS and other consumables not listed under PBS. In some States and Territories such as ACT, patients actually pay more out-of-pocket costs under optimal care (Option 1) than usual care. The cause lies in how care is funded in each State and Territory. According to the internet survey [2], in the smallest territory ACT where Canberra, Australia’s capital city is situated, patients may have out-of-pocket costs for medicines but do not pay for dressings and other consumables including compression bandages. Thus, even under usual care, the out-of-pocket costs are low in ACT and would be higher under optimal care for Option 1 where patients in specialist wound clinics may have out-of-pocket expenses for certain consumables that would not be incurred under Option 2 arrangements. However, the proportion of consumables paid by patients in different States and Territories (Additional file 1: Table S3) reported in the internet survey [2] might not be accurate due to the small sample size and low response rate. Therefore, findings on patients’ out-of-pocket expenditure presented in this study should be interpreted with caution.

### Limitations

There are several limitations in this study. Firstly, data on clinical effectiveness such as healing rate was sourced from a non-Australian study, although it is reassuring that a study in Queensland, Australia, reported similar healing rates with implementation of evidence-based wound care [5]. We assumed that 50% of patients in the usual care group were receiving compression therapy across all jurisdictions and that the same healing, recurrence and hospitalisation rates applied to all States and Territories in Australia due to lack of local data. However, as the health service pathways and use of compression therapy differ between States and Territories, the probability of healing, recurrence and hospitalisation may also vary. We also assumed constant risk of recurrence over time in the absence of long-term follow up data, and in order to avoid overestimation, we limited the time horizon to 5 years. The model inputs can be updated when national wound registry data become available in Australia. Secondly, we relied on published literature to estimate hospitalisation rate among patients treated with optimal care. In Australia, the number of hospitalisations attributed to VLUs can be obtained from either International Classification of Diseases (ICD) codes [29] or AR-DRG codes [23]. But whether patients receive optimal care or usual care prior to hospitalisation is not known. A longitudinal cohort study that follows patients over time would be helpful to determine the hospitalisation rate between treatment groups. It is also important to note that in this analysis both optimal care and usual care are simplified and extreme scenarios for modelling while current practice in Australia is probably a mixture of optimal care and usual care. We found that if usual care was taken as ongoing national practice, the number of prevalent VLU cases and hospitalisations would rocket as a result of increasing new cases and non-healing cases over time. If optimal care was implemented, then a great reduction in VLU-related hospitalisation would be expected. Thirdly, in the absence of Australian data, the annual incidence rate of VLUs among general population aged 60 and over of 1.2 per 100 person-years was derived from a UK study where a prevalence of 1.69% was reported [18]. In our study, we used this incidence rate with a prevalence of 0.33% instead but did not check the consistency of these epidemiological parameters [30]. Moreover, the prevalence estimate of 0.33% used in the baseline analysis was sourced from a study conducted in the 1990s and is very likely to be an underestimate of current prevalence of VLU. The scenario analysis showed that higher prevalence is associated with greater cost savings. Thus, total cost savings to the Australian society may have been underestimated in our baseline analysis. Fourthly, the costs of consultation with GP, nurse practitioner and vascular surgeon were assumed to follow the scheduled fees listed on MBS, while in fact, patients sometimes pay an additional fee for the consultations. Thus, the out-of-pocket cost in this study may have been underestimated. Fifth, although Australian government and State and Territory governments share the funding responsibility for community nursing services [27], the proportion of this share is not well documented. We assumed that the Australian government covered all costs of community nursing services. Thus, the total costs borne by State government may have been underestimated in this analysis. We also assumed that all hospitalisations as a result of VLU complications would occur in public hospitals and hence health system costs may have been overestimated and out-of-pocket costs to individuals underestimated as some of the patients have private health insurance and may be hospitalised for VLUs in private hospitals. Finally, the cost-savings presented in this study need to be interpreted with caution. Due to lack of data on lost productivity, we had to assume people aged 60 years and over are retired. With optimal care, patients are expected to heal faster and have fewer recurrent ulcers which means that there will be less lost productivity compared with usual care. Thus, excluding cost estimation on lost productivity could underestimate cost-savings to society. We also assumed that under optimal care, compression therapy would be provided by trained health professionals, but we did not take into account the cost of education and training of health care providers in evidence-based wound management. The costs of doing a full implementation of guideline-based wound care would be higher than the costs included in this study. As a result, the cost-savings to the health system could be overestimated.

## Conclusion

As the Australian population ages, the prevalence of VLUs will continue to rise. While Australia prides itself in having one of the developed world’s best health care systems, and does very well compared to the rest of the world in terms of health, wound management is an area where Australia trails behind. Implementing a large-scale change to current practice is complex and takes time, but this study provides important evidence that adequate reimbursement for guideline-based services and products would not only result in cost savings for patients and the health system but also improve health outcomes and quality of life for patients with VLUs. Therefore, we recommend better reimbursement of guideline-based care for VLUs by the Australian health system. Measures could include listing compression therapy in PBS for all affected individuals or creating wound-specific MBS items. We hope that this study could be of great relevance and importance to other countries that face similar issues and could benefit from investing in guideline-based wound care.

## Additional file

Additional file 1: **Table S1.** Key elements of guideline-based optimal care*.* **Table S2.** General population aged 60 years and older, population growth and VLU-affected population in Australia*.* **Table S3.** Proportion of patients treated by health service provider and proportion of consumables paid by patients in Australia*.* **Table S4.** Markov model cost inputs for all States and Territories (AUD 2015 price)*.* **Table S5.** Estimated number of people 60 years and older affected by VLU and hospitalisations over 5 years in Australia*.* **Table S6.** Baseline outcomes for all affected persons over 5 years by State and Territory (optimal care service delivery option 1)*.* **Table S7.** Baseline outcomes for all affected persons over 5 years by State and Territory (optimal care service delivery option 2)*.* **Table S8.** Expected costs and QALYs per person over 5 years (130 cycles) if the person entered the model from cycle 0 (Optimal care service delivery option 1, AUD 2015 prices)*.* **Table S9.** Expected costs and QALYs per person over 5 years (130 cycles) if the person entered the model from cycle 0 (Optimal care service delivery option 2, AUD 2015 prices)*.* **Table S10.** Distribution of cost savings to the Australian government and State and Territory government*.* **Table S11.** CHEERS checklist*.* **Figure S1.** Probabilistic sensitivity analysis results for VIC*.* **Figure S2.** Probabilistic sensitivity analysis results for QLD*.* **Figure S3.** Probabilistic sensitivity analysis results for SA*.* **Figure S4.** Probabilistic sensitivity analysis results for WA*.* **Figure S5.** Probabilistic sensitivity analysis results for NT **Figure S6.** Probabilistic sensitivity analysis results for TAS*.* **Figure S7.** Probabilistic sensitivity analysis results for ACT*.* (DOCX 1672 kb)

## Abbreviations

ABPI: Ankle-brachial pressure index
ACT: Australian Capital Territory
AR-DRG: Australian Refined Diagnosis Related Groups
GP: General Practitioner
ICD: International Classification of Diseases
ICER: Incremental cost-effectiveness ratio
IHPA: Independent Hospital Pricing Authority
MBS: Medicare Benefits Schedule
NSW: New South Wales
NT: Northern Territory
PBS: Pharmaceutical Benefits Scheme
QALY: Quality-adjusted life year
QLD: Queensland
QoL: Quality of life
RPBS: Repatriation Schedule of Pharmaceutical Benefits
SA: South Australia
TAS: Tasmania
VIC: Victoria
VLU: Venous leg ulcer
WA: Western Australia
