# Supplementary material for: Cost-effectiveness analysis of guideline-based optimal care for venous leg ulcers in Australia
Source: BMC Health Serv Res. 2018 Jun 7;18:421. doi: 10.1186/s12913-018-3234-3 (PMC5992639; doi:10.1186/s12913-018-3234-3)
Supplement: Supplementary file 1 — Table S1. Key elements of guideline-based optimal care. Table S2. General population aged 60 years and older, population growth and VLU-affected population in Australia. Table S3. Proportion of patients treated by health service provider and proportion of consumables paid by patients in Australia. Table S4. Markov model cost inputs for all States and Territories (AUD 2015 price). Table S5. Estimated number of people 60 years and older affected by VLU and hospitalisations over 5 years in Australia. Table S6. Baseline outcomes for all affected persons over 5 years by State and Territory (optimal care service delivery option 1). Table S7. Baseline outcomes for all affected persons over 5 years by State and Territory (optimal care service delivery option 2). Table S8. Expected costs and QALYs per person over 5 years (130 cycles) if the person entered the model from cycle 0 (Optimal care service delivery option 1, AUD 2015 prices). Table S9. Expected costs and QALYs per person over 5 years (130 cycles) if the person entered the model from cycle 0 (Optimal care service delivery option 2, AUD 2015 prices). Table S10. Distribution of cost savings to the Australian government and State and Territory government. Table S11. CHEERS checklist. Figure S1. Probabilistic sensitivity analysis results for VIC. Figure S2. Probabilistic sensitivity analysis results for QLD. Figure S3. Probabilistic sensitivity analysis results for SA. Figure S4. Probabilistic sensitivity analysis results for WA. Figure S5. Probabilistic sensitivity analysis results for NT Figure S6. Probabilistic sensitivity analysis results for TAS. Figure S7. Probabilistic sensitivity analysis results for ACT. (DOCX 1672 kb) [file 12913_2018_3234_MOESM1_ESM.docx]

**Appendix**

Table S1. Key elements of guideline-based optimal care

| Conditions | Key procedures |
| --- | --- |
| Uncomplicated VLU | - comprehensive assessment by health professional trained in the assessment and management of VLUs - patients undergo an initial ankle-brachial pressure index (ABPI)/or vascular assessment - provide patients with appropriate education on their condition and its management - high level (30mmHg and above) compression therapy |
| Infected VLU | - comprehensive assessment by health professional trained in the assessment and management of VLUs - pathology to confirm bacteria - debridement - infection management with use of topical antimicrobials and/or oral antibiotics |
| Healed VLU | Moderate compression (20mmHg and above) will be applied after healing to prevent recurrence and patient education by health professional trained in the assessment and management of VLUs. |

Demographic details in each State and Territory in Australia

The number of general population aged 60 years and over starting from ‘No VLU’ state in 2015 differed between States and Territories ([1](#_ENREF_1)) and was summarized in Table A2. Growth rate in population aged 60 and over every fortnight in each state was derived using population growth between years 2015 and 2016 ([1](#_ENREF_1)) and assuming this growth rate remained constant over duration of the model (5 years). The population affected with VLU in 2015 was derived by multiplying the population in that age group ([1](#_ENREF_1)) by the only reported prevalence of VLUs among people aged 60 and over in Australia (0.33%) from a study conducted in the 1990s ([2](#_ENREF_2)).

Table S2. General population aged 60 years and older, population growth and VLU-affected population in Australia

|  | NSW | VIC | QLD | SA | WA | NT | TAS | ACT | Australia* | Source |
| --- | --- | --- | --- | --- | --- | --- | --- | --- | --- | --- |
| Population 60+ in 2015 | 1,600,311 | 1,215,981 | 942,306 | 395,187 | 465,725 | 26,796 | 128,038 | 66,095 | 4,840,439 | ([1](#_ENREF_1)) |
| Population 60+ in 2016 | 1,639,438 | 1,253,534 | 974,058 | 404,375 | 479,946 | 27,703 | 131,347 | 68,193 | 4,978,594 | ([1](#_ENREF_1)) |
| Estimated Population growth per fortnight | 0.20% | 0.22% | 0.22% | 0.20% | 0.21% | 0.21% | 0.21% | 0.20% | 0.21% | calculated |
| Affected with VLU in 2015** | 5,281 | 4,013 | 3,110 | 1,304 | 1,537 | 88 | 423 | 218 | 15,973 | calculated |
| *Total population in Australia includes population in external territories.  ** Based on prevalence of 0.33% | | | | | | | | | | |

Resources use and costs

*Usual care*

According to a survey that aimed to capture treatment variations across jurisdictions in Australia ([3](#_ENREF_3)), 95% of patients were firstly diagnosed by a general practitioner (GP). Therefore, we assumed that under usual care, patients with unhealed VLUs would receive a one-off initial assessment by a GP. The internet survey also reported that patients would receive treatment at GP clinics, by community nursing services or outpatient clinics and the proportions of patients treated in these three settings would vary by State and Territory in Australia (Table A3). In this study, we assumed that patients would receive treatment by GP, community nurse or outpatient clinicians twice a week. Consultations with GP and vascular surgeon were valued in line with Australian Federal Government reimbursements through MBS. Consultation with community nurse was estimated at a wage rate of Registered Nurse ([4-11](#_ENREF_4)). We assumed no additional fees or out-of-pocket costs for consultations in this analysis. Patients with unhealed VLUs were also assumed to receive absorbent dressing changes twice a week and, given that 40-60% of VLUs in Australia did not receive adequate compression therapy ([12](#_ENREF_12), [13](#_ENREF_13)), we assumed that 50% of patients in the usual care group were receiving compression therapy. It was also assumed that patients in the usual care group would pay for a proportion of consumables including compression therapy and this differed across States and Territories (Table A3). If patients developed an infected ulcer, they were assumed to receive bacterial wound swab to determine the pathogen that caused infection every week and received systemic antibiotics. Moreover, the frequency of clinical visits and dressing changes were assumed to increase to three times a week with an infection. The unit cost of compression therapy and medication prescribed was informed by prices listed on PBS with some out-of-pocket costs to patients. For example using PBS data, if the actual cost of the medicine (dispensed price for maximum quantity), exceeded the general patient co-payment of $38.80 (amount current as of 1 January 2017), then the additional government subsidy was included as a health system cost (government subsidy=actual cost- $38.80 cost to patient). This patient cost of $38.80 was included as an out-of-pocket cost. If a pharmaceutical item had a dispensed price for maximum quantity less than the general patient co-payment of $38.80, then the patient pays the dispensed price and government subsidies do not apply. We assumed that all patients were general patients (non-concession) and we also did not include the additional discretionary fee to general patients that pharmacists may charge if item has a dispensed price less than the general patient co-payment of $38.80. A review of market prices for all relevant products was used to value all consumable items that are not reimbursed by MBS or PBS. Once the ulcer healed, patients would receive no prevention care. The hospitalisation cost was informed by the National Hospital Cost Data Collection Australian Public Hospitals Cost Report 2014–2015, Round 19 ([14](#_ENREF_14)). The costs of treatment for each health state in usual care group were calculated for each State and Territory in Australia in Appendix Table A4.

*Optimal care service delivery option 1: Specialist Wound Clinic with Nurse Practitioner and vascular surgeon*

Following guideline recommendations, patients with unhealed VLUs were assumed to receive a one-off initial ABPI/or vascular assessment by a nurse practitioner and a vascular surgeon and then undergo medical checks by nurse practitioner every week. The costs of consultation to nurse practitioner and ABPI assessment were informed by MBS items. In addition to weekly dressing changes, patients were also prescribed high compression therapy every week. If the ulcer became infected, patients would receive a one-off bacterial wound swab, conservative sharp wound debridement every week and systemic antibiotics. Once the ulcer was healed, patients would continue to be cared by nurse practitioners and receive moderate compression therapy with clinic visits every three months and education for prevention. Product costs of compression therapy were assumed to be covered by the health system in the optimal care group (both options 1 and 2) but patients would still have some out of pocket costs for other consumables such as antibiotics under optimal care. Cost of patient education in optimal care would be covered by service costs of a consultation. Cost of setting up a specialist wound clinic and equipment costs were not included in this analysis.

*Optimal care service delivery option 2: GP, community nurses, outpatient clinics*

Patients with unhealed VLUs were assumed to receive a one-off initial ABPI assessment by a GP and then undergo medical checks by GP, community nurse or outpatient clinician with wound expertise every week. All patients receive evidence-based treatment and prevention of VLU as per option 1 with product costs of compression therapy assumed to be covered by the health system but service delivery costs were calculated using the proportions of diagnosed patients being treated at GP, community nurse or outpatient clinics by State and Territory presented in Table A3. We did not include additional service costs for applying compression therapy or extra time for these consultations. Costs of education and training of health professionals in wound management were not included in this analysis.

Table S3. Proportion of patients treated by health service provider and proportion of consumables paid by patients in Australia

|  | NSW | VIC | QLD | SA | WA | NT | TAS | ACT | Source |
| --- | --- | --- | --- | --- | --- | --- | --- | --- | --- |
| Community care |  |  |  |  |  |  |  |  |  |
| Proportion of diagnosed patients being treated in community care | 68.0% | 60.0% | 3.0% | 95.0% | 55.0% | 95.0% | 40.0% | 70.0% | ([3](#_ENREF_3)) |
| Proportion of consumable costs paid by the patient in community nursing | 5.0% | 90.0% | 100.0% | 0.0% | 0.0% | 0.0% | 30.0% | 0.0% | ([3](#_ENREF_3)) |
| GP |  |  |  |  |  |  |  |  |  |
| Proportion of diagnosed patients being treated at a GP clinic | 12.0% | 30.0% | 92.0% | 5.0% | 15.0% | 1.0% | 35.0% | 0.0% | ([3](#_ENREF_3)) |
| Proportion of consumable costs paid by the patient at GP clinics | 60.0% | 85.0% | 95.0% | 88.0% | 100.0% | 88.0% | 100.0% | 0.0% | ([3](#_ENREF_3)) |
| Outpatient clinic* |  |  |  |  |  |  |  |  |  |
| Proportion of diagnosed patients being treated at an outpatient clinic | 20.0% | 10.0% | 5.0% | 0.0% | 30.0% | 4.0% | 25.0% | 30.0% | ([3](#_ENREF_3)) |
| * We assumed that there was no out-of-pocket cost for consumables in outpatient clinic. | | | | | | | | | |

Table S4. Markov model cost inputs for all States and Territories (AUD 2015 price)

|  | **NSW** | **VIC** | | **QLD** | **SA** | **WA** | | **NT** | **TAS** | **ACT** |
| --- | --- | --- | --- | --- | --- | --- | --- | --- | --- | --- |
| **Ongoing costs for health states (fortnightly)** | | | | | | | | | | |
| ***Usual care*** |  | |  |  |  | |  |  |  |  |
| Healed VLU | $0.00 | | $0.00 | $0.00 | $0.00 | | $0.00 | $0.00 | $0.00 | $0.00 |
| Unhealed VLU | $226.34 | | $234.54 | $368.24 | $136.12 | | $262.04 | $162.31 | $291.18 | $227.05 |
| ***Optimal care (Option 1: Specialist Wound Clinic with Nurse Practitioner)^a,b^*** | | | | | | | | | | |
| Healed VLU | $26.05 | | $26.05 | $26.05 | $26.05 | | $26.05 | $26.05 | $26.05 | $26.05 |
| Service costs | $9.76 | | $9.76 | $9.76 | $9.76 | | $9.76 | $9.76 | $9.76 | $9.76 |
| Cost of compression | $16.29 | | $16.29 | $16.29 | $16.29 | | $16.29 | $16.29 | $16.29 | $16.29 |
| Unhealed VLU | $244.07 | | $244.07 | $244.07 | $244.07 | | $244.07 | $244.07 | $244.07 | $244.07 |
| Service costs* | $117.10 | | $117.10 | $117.10 | $117.10 | | $117.10 | $117.10 | $117.10 | $117.10 |
| Costs of compression* | $102.00 | | $102.00 | $102.00 | $102.00 | | $102.00 | $102.00 | $102.00 | $102.00 |
| ***Optimal care (Option 2: GP, community nursing, outpatient clinics)^a,b,c^*** | | | | | | | | | | |
| Healed VLU | $28.24 | | $28.24 | $28.24 | $28.24 | | $28.24 | $28.24 | $28.24 | $28.24 |
| Service costs | $11.95 | | $11.95 | $11.95 | $11.95 | | $11.95 | $11.95 | $11.95 | $11.95 |
| Cost of compression | $16.29 | | $16.29 | $16.29 | $16.29 | | $16.29 | $16.29 | $16.29 | $16.29 |
| Unhealed VLU | $202.25 | | $205.33 | $268.58 | $157.64 | | $220.08 | $170.44 | $233.54 | $203.41 |
| Service costs* | $75.28 | | $78.36 | $141.62 | $30.67 | | $93.11 | $43.48 | $106.57 | $76.45 |
| Costs of compression* | $102.00 | | $102.00 | $102.00 | $102.00 | | $102.00 | $102.00 | $102.00 | $102.00 |
| **Initial costs for Unhealed VLU** | | | | | | | | | | |
| ***Usual care*** | $71.70 | | $71.70 | $71.70 | $71.70 | | $71.70 | $71.70 | $71.70 | $71.70 |
| ***Optimal care (Option 1)*** | $190.74 | | $0.00 | $0.00 | $0.00 | | $0.00 | $190.74 | $0.00 | $0.00 |
| ***Optimal care (Option 2)*** | $137.14 | | $0.00 | $0.00 | $0.00 | | $0.00 | $137.14 | $0.00 | $0.00 |
| ^a^ Under optimal care consumable costs of compression therapy are subsidised through the health system  ^b^ Costs of education and training of health professionals in wound management not included;  ^c^ Costs of education and training of health professionals in wound management and cost of establishing wound clinics not included  * Service costs and cost of compression do not add up to total costs for health states because the total cost includes other products (such as silver dressings, antibiotics). | | | | | | | | | | |

Table S5. Estimated number of people 60 years and older affected by VLU and hospitalisations over 5 years in Australia

|  | NSW | VIC | QLD | SA | WA | NT | TAS | ACT | Australia |
| --- | --- | --- | --- | --- | --- | --- | --- | --- | --- |
| Affected with VLU at baseline | 5,281 | 4,013 | 3,110 | 1,304 | 1,537 | 88 | 423 | 218 | 15,973 |
| New VLU cases over 5 years | 100,678 | 77,733 | 60,659 | 24,788 | 29,745 | 1,725 | 8,084 | 4,233 | 307,646 |
| Affected by VLU over 5 years | 105,959 | 81,746 | 63,769 | 26,092 | 31,282 | 1,814 | 8,507 | 4,451 | 323,619 |
| Hospitalisations under usual care over 5 years | 7,594 | 5,843 | 4,557 | 1,870 | 2,239 | 130 | 607 | 320 | 23,160 |
| Hospitalisations under optimal care over 5 years | 486 | 374 | 292 | 120 | 143 | 8 | 39 | 20 | 1,483 |
| Hospitalisations avoided | 7,108 | 5,469 | 4,265 | 1,751 | 2,096 | 122 | 568 | 300 | 21,677 |

Table S6. Baseline outcomes for all affected persons over 5 years by State and Territory (optimal care service delivery option 1)

|  | NSW | VIC | QLD | SA | WA | NT | TAS | ACT |
| --- | --- | --- | --- | --- | --- | --- | --- | --- |
| Total costs in usual care group | $842,208,404 | $667,655,044 | $771,918,979 | $137,802,310 | $281,235,478 | $11,000,897 | $83,600,586 | $35,584,954 |
| Total costs in optimal care group | $393,551,203 | $302,898,487 | $236,198,200 | $96,930,610 | $116,035,260 | $6,745,645 | $31,494,779 | $16,573,752 |
| Total incremental costs | -$448,657,200 | -$364,756,558 | -$535,720,779 | -$40,871,700 | -$165,200,218 | -$4,255,252 | -$52,105,806 | -$19,011,202 |
|  |  |  |  |  |  |  |  |  |
| Health system costs in usual care group | $800,189,957 | $539,557,238 | $660,220,523 | $130,212,334 | $266,506,128 | $10,581,548 | $74,980,773 | $34,621,402 |
| Health system costs in optimal care group | $370,093,304 | $284,834,564 | $222,111,441 | $91,153,178 | $109,117,265 | $6,343,795 | $29,615,925 | $15,586,673 |
| Incremental costs | -$430,096,652 | -$254,722,673 | -$438,109,083 | -$39,059,156 | -$157,388,863 | -$4,237,754 | -$45,364,848 | -$19,034,729 |
|  |  |  |  |  |  |  |  |  |
| Costs by Australian government in usual care group | $728,705,737 | $484,573,321 | $617,349,471 | $112,604,886 | $245,437,420 | $9,356,016 | $69,264,390 | $31,609,507 |
| Costs by Australian government in optimal care group | $365,450,732 | $281,260,159 | $219,324,253 | $90,009,720 | $107,748,363 | $6,264,289 | $29,244,084 | $15,391,368 |
| Incremental costs | -$363,255,006 | -$203,313,162 | -$398,025,218 | -$22,595,166 | -$137,689,057 | -$3,091,728 | -$40,020,305 | -$16,218,139 |
|  |  |  |  |  |  |  |  |  |
| Costs by State and Territory government in usual care group | $71,484,219 | $54,983,916 | $42,871,053 | $17,607,447 | $21,068,708 | $1,225,532 | $5,716,383 | $3,011,895 |
| Costs by State and Territory government in optimal care group | $4,642,573 | $3,574,406 | $2,787,188 | $1,143,457 | $1,368,902 | $79,506 | $371,840 | $195,305 |
| Incremental costs | -$66,841,647 | -$51,409,511 | -$40,083,865 | -$16,463,990 | -$19,699,805 | -$1,146,026 | -$5,344,542 | -$2,816,590 |
|  |  |  |  |  |  |  |  |  |
| Out-of-pocket costs in usual care group | $42,018,447 | $128,097,807 | $111,698,455 | $7,589,976 | $14,729,350 | $419,349 | $8,619,813 | $963,552 |
| Out-of-pocket costs in optimal care group | $23,457,899 | $18,063,922 | $14,086,759 | $5,777,432 | $6,917,995 | $401,850 | $1,878,855 | $987,080 |
| Incremental costs | -$18,560,548 | -$110,033,884 | -$97,611,697 | -$1,812,544 | -$7,811,355 | -$17,498 | -$6,740,959 | $23,528 |
|  |  |  |  |  |  |  |  |  |
| Total costs of compression products in usual care group^a^ | $159,826,014 | $122,949,131 | $95,869,402 | $39,366,135 | $47,111,750 | $2,740,698 | $12,780,786 | $6,735,356 |
| Total costs of compression products in optimal care group^b^ | $176,982,534 | $136,179,721 | $106,188,668 | $43,591,172 | $52,174,967 | $3,034,222 | $14,157,931 | $7,455,859 |
| Incremental costs | $17,156,520 | $13,230,590 | $10,319,266 | $4,225,037 | $5,063,217 | $293,524 | $1,377,145 | $720,502 |
|  |  |  |  |  |  |  |  |  |
| Costs of compression products in usual care group indirectly covered by health system | $142,884,456 | $25,204,572 | $9,203,463 | $37,634,025 | $40,044,988 | $2,716,580 | $6,773,817 | $6,735,356 |
| Total of compression products in optimal care group covered by health system | $176,982,534 | $136,179,721 | $106,188,668 | $43,591,172 | $52,174,967 | $3,034,222 | $14,157,931 | $7,455,859 |
| Incremental costs | $34,098,078 | $110,975,149 | $96,985,206 | $5,957,147 | $12,129,980 | $317,642 | $7,384,114 | $720,502 |
|  |  |  |  |  |  |  |  |  |
| Costs of other dressings in usual care group | $12,384,949 | $9,573,157 | $7,482,513 | $3,079,821 | $3,633,147 | $214,011 | $988,005 | $519,415 |
| Costs of other dressings in optimal care group | $22,535,704 | $17,353,779 | $13,532,969 | $5,550,305 | $6,646,029 | $386,053 | $1,804,992 | $948,275 |
| Incremental costs | $10,150,755 | $7,780,622 | $6,050,456 | $2,470,484 | $3,012,882 | $172,042 | $816,987 | $428,860 |
|  |  |  |  |  |  |  |  |  |
| Total QALYs in usual care group | 156,162 | 120,075 | 93,624 | 38,465 | 46,022 | 2,679 | 12,479 | 6,586 |
| Total QALYs in optimal care group | 165,458 | 127,222 | 99,197 | 40,754 | 48,761 | 2,839 | 13,221 | 6,978 |
| Incremental QALYs | 9,296 | 7,148 | 5,573 | 2,290 | 2,740 | 159 | 743 | 392 |

Table S7. Baseline outcomes for all affected persons over 5 years by State and Territory (optimal care service delivery option 2)

|  | NSW | VIC | QLD | SA | WA | NT | TAS | ACT |
| --- | --- | --- | --- | --- | --- | --- | --- | --- |
| Total costs in usual care group | $842,208,404 | $667,655,044 | $771,918,979 | $137,802,310 | $281,235,478 | $11,000,897 | $83,600,586 | $35,584,954 |
| Total costs in optimal care group | $357,393,669 | $277,287,157 | $252,242,934 | $77,607,342 | $110,353,295 | $5,609,855 | $30,972,615 | $15,099,923 |
| Total incremental costs | -$484,814,735 | -$390,367,888 | -$519,676,045 | -$60,194,967 | -$170,882,183 | -$5,391,042 | -$52,627,971 | -$20,485,031 |
|  |  |  |  |  |  |  |  |  |
| Health system costs in usual care group | $800,189,957 | $539,557,238 | $660,220,523 | $130,212,334 | $266,506,128 | $10,581,548 | $74,980,773 | $34,621,402 |
| Health system costs in optimal care group | $354,082,689 | $262,780,759 | $239,455,340 | $77,136,002 | $109,084,425 | $5,590,660 | $30,050,406 | $15,061,119 |
| Incremental costs | -$446,107,268 | -$276,776,479 | -$420,765,183 | -$53,076,332 | -$157,421,703 | -$4,990,889 | -$44,930,366 | -$19,560,283 |
|  |  |  |  |  |  |  |  |  |
| Costs by Australian government in usual care group | $728,705,737 | $484,573,321 | $617,349,471 | $112,604,886 | $245,437,420 | $9,356,016 | $69,264,390 | $31,609,507 |
| Costs by Australian government in optimal care group | $349,440,116 | $259,206,353 | $236,668,152 | $75,992,545 | $107,715,523 | $5,511,154 | $29,678,566 | $14,865,814 |
| Incremental costs | -$379,265,621 | -$225,366,968 | -$380,681,318 | -$36,612,342 | -$137,721,897 | -$3,844,863 | -$39,585,824 | -$16,743,693 |
|  |  |  |  |  |  |  |  |  |
| Costs by State and Territory government in usual care group | $71,484,219 | $54,983,916 | $42,871,053 | $17,607,447 | $21,068,708 | $1,225,532 | $5,716,383 | $3,011,895 |
| Costs by State and Territory government in optimal care group | $4,642,573 | $3,574,406 | $2,787,188 | $1,143,457 | $1,368,902 | $79,506 | $371,840 | $195,305 |
| Incremental costs | -$66,841,647 | -$51,409,511 | -$40,083,865 | -$16,463,990 | -$19,699,805 | -$1,146,026 | -$5,344,542 | -$2,816,590 |
|  |  |  |  |  |  |  |  |  |
| Out-of-pocket costs in usual care group | $42,018,447 | $128,097,807 | $111,698,455 | $7,589,976 | $14,729,350 | $419,349 | $8,619,813 | $963,552 |
| Out-of-pocket costs in optimal care group | $3,310,980 | $14,506,398 | $12,787,594 | $471,340 | $1,268,870 | $19,195 | $922,209 | $38,805 |
| Incremental costs | -$38,707,467 | -$113,591,409 | -$98,910,862 | -$7,118,636 | -$13,460,480 | -$400,154 | -$7,697,604 | -$924,747 |
|  |  |  |  |  |  |  |  |  |
| Total costs of compression products in usual care group^a^ | $159,826,014 | $122,949,131 | $95,869,402 | $39,366,135 | $47,111,750 | $2,740,698 | $12,780,786 | $6,735,356 |
| Total costs of compression products in optimal care group^b^ | $176,982,534 | $136,179,721 | $106,188,668 | $43,591,172 | $52,174,967 | $3,034,222 | $14,157,931 | $7,455,859 |
| Incremental costs | $17,156,520 | $13,230,590 | $10,319,266 | $4,225,037 | $5,063,217 | $293,524 | $1,377,145 | $720,502 |
|  |  |  |  |  |  |  |  |  |
| Costs of compression products in usual care group indirectly covered by health system | $142,884,456 | $25,204,572 | $9,203,463 | $37,634,025 | $40,044,988 | $2,716,580 | $6,773,817 | $6,735,356 |
| Total costs of compression products in optimal care group covered by health system | $176,982,534 | $136,179,721 | $106,188,668 | $43,591,172 | $52,174,967 | $3,034,222 | $14,157,931 | $7,455,859 |
| Incremental costs | $34,098,078 | $110,975,149 | $96,985,206 | $5,957,147 | $12,129,980 | $317,642 | $7,384,114 | $720,502 |
|  |  |  |  |  |  |  |  |  |
| Costs of other dressings in usual care group | $12,384,949 | $9,573,157 | $7,482,513 | $3,079,821 | $3,633,147 | $214,011 | $988,005 | $519,415 |
| Costs of other dressings in optimal care group | $22,535,704 | $17,353,779 | $13,532,969 | $5,550,305 | $6,646,029 | $386,053 | $1,804,992 | $948,275 |
| Incremental costs | $10,150,755 | $7,780,622 | $6,050,456 | $2,470,484 | $3,012,882 | $172,042 | $816,987 | $428,860 |
|  |  |  |  |  |  |  |  |  |
| Total QALYs in usual care group | 156,162 | 120,075 | 93,624 | 38,465 | 46,022 | 2,679 | 12,479 | 6,586 |
| Total QALYs in optimal care group | 165,458 | 127,222 | 99,197 | 40,754 | 48,761 | 2,839 | 13,221 | 6,978 |
| Incremental QALYs | 9,296 | 7,148 | 5,573 | 2,290 | 2,740 | 159 | 743 | 392 |

Table S8. Expected costs and QALYs per person over 5 years (130 cycles) if the person entered the model from cycle 0 (Optimal care service delivery option 1, AUD 2015 prices)

|  | NSW | VIC | QLD | SA | WA | NT | TAS | ACT | Australia |
| --- | --- | --- | --- | --- | --- | --- | --- | --- | --- |
| Total costs in usual care group | $18,098 | $18,663 | $27,671 | $12,027 | $20,511 | $13,796 | $22,472 | $18,149 | $19,962 |
| Total costs in optimal care group | $9,424 | $9,437 | $9,441 | $9,422 | $9,434 | $9,432 | $9,433 | $9,427 | $9,432 |
| Total incremental costs | -$8,674 | -$9,226 | -$18,229 | -$2,605 | -$11,077 | -$4,364 | -$13,039 | -$8,722 | -$10,530 |
|  |  |  |  |  |  |  |  |  |  |
| Health system costs in usual care group | $17,196 | $15,085 | $23,669 | $11,365 | $19,438 | $13,271 | $20,157 | $17,658 | $17,747 |
| Health system costs in optimal care group | $8,798 | $8,810 | $8,814 | $8,796 | $8,807 | $8,805 | $8,806 | $8,801 | $8,805 |
| Incremental costs | -$8,398 | -$6,276 | -$14,855 | -$2,569 | -$10,631 | -$4,465 | -$11,351 | -$8,857 | -$8,942 |
|  |  |  |  |  |  |  |  |  |  |
| Costs by Australian government in usual care group | $15,674 | $13,563 | $22,146 | $9,844 | $17,916 | $11,749 | $18,634 | $16,136 | $16,225 |
| Costs by Australian government in optimal care group | $8,674 | $6,680 | $6,432 | $8,402 | $8,168 | $8,493 | $7,428 | $8,508 | $7,622 |
| Incremental costs | -$7,000 | -$6,882 | -$15,714 | -$1,441 | -$9,747 | -$3,256 | -$11,206 | -$7,628 | -$8,603 |
|  |  |  |  |  |  |  |  |  |  |
| Costs by State and Territory government in usual care group | $1,522 | $1,523 | $1,523 | $1,522 | $1,522 | $1,522 | $1,523 | $1,522 | $1,522 |
| Costs by State and Territory government in optimal care group | $124 | $2,129 | $2,382 | $394 | $639 | $313 | $1,378 | $292 | $1,183 |
| Incremental costs | -$1,398 | $607 | $859 | -$1,128 | -$884 | -$1,209 | -$144 | -$1,230 | -$339 |
|  |  |  |  |  |  |  |  |  |  |
| Out-of-pocket costs in usual care group | $902 | $3,578 | $4,002 | $662 | $1,073 | $525 | $2,315 | $491 | $2,215 |
| Out-of-pocket costs in optimal care group | $626 | $627 | $628 | $626 | $627 | $627 | $627 | $626 | $627 |
| Incremental costs | -$276 | -$2,950 | -$3,374 | -$36 | -$446 | $102 | -$1,688 | $135 | -$1,588 |
|  |  |  |  |  |  |  |  |  |  |
| Total costs of compression products in usual care group^a^ | $3,431 | $3,434 | $3,435 | $3,431 | $3,433 | $3,433 | $3,433 | $3,432 | $3,433 |
| Total costs of compression products in optimal care group^b^ | $4,047 | $4,051 | $4,053 | $4,046 | $4,050 | $4,050 | $4,050 | $4,048 | $4,049 |
| Incremental costs | $615 | $617 | $618 | $615 | $617 | $617 | $617 | $616 | $617 |
|  |  |  |  |  |  |  |  |  |  |
| Total QALYs in usual care group | 3.04 | 3.04 | 3.04 | 3.04 | 3.04 | 3.04 | 3.04 | 3.04 | 3.04 |
| Total QALYs in optimal care group | 3.22 | 3.22 | 3.22 | 3.22 | 3.22 | 3.22 | 3.22 | 3.22 | 3.22 |
| Incremental QALYs | 0.18 | 0.18 | 0.18 | 0.18 | 0.18 | 0.18 | 0.18 | 0.18 | 0.18 |
| a Costs of compression therapy in usual care group were covered by covered by out-of-pocket expenditure and government’s funding to health professionals.  b Costs of compression therapy in optimal care group were covered by Australian government. | | | | | | | | | |

Table S9. Expected costs and QALYs per person over 5 years (130 cycles) if the person entered the model from cycle 0 (Optimal care service delivery option 2, AUD 2015 prices)

|  | NSW | VIC | QLD | SA | WA | NT | TAS | ACT | Australia |
| --- | --- | --- | --- | --- | --- | --- | --- | --- | --- |
| Total costs in usual care group | $18,098 | $18,663 | $27,671 | $12,027 | $20,511 | $13,796 | $22,472 | $18,149 | $19,962 |
| Total costs in optimal care group | $8,367 | $8,456 | $10,064 | $7,237 | $8,827 | $7,567 | $9,167 | $8,399 | $8,694 |
| Total incremental costs | -$9,731 | -$10,207 | -$17,606 | -$4,790 | -$11,684 | -$6,228 | -$13,305 | -$9,750 | -$11,268 |
|  |  |  |  |  |  |  |  |  |  |
| Health system costs in usual care group | $17,196 | $15,085 | $23,669 | $11,365 | $19,438 | $13,271 | $20,157 | $17,658 | $17,747 |
| Health system costs in optimal care group | $8,279 | $7,952 | $9,495 | $7,186 | $8,712 | $7,538 | $8,860 | $8,374 | $8,402 |
| Incremental costs | -$8,917 | -$7,133 | -$14,174 | -$4,180 | -$10,726 | -$5,733 | -$11,297 | -$9,283 | -$9,345 |
|  |  |  |  |  |  |  |  |  |  |
| Costs by Australian government in usual care group | $15,674 | $13,563 | $22,146 | $9,844 | $17,916 | $11,749 | $18,634 | $16,136 | $16,225 |
| Costs by Australian government in optimal care group | $8,155 | $5,822 | $7,113 | $6,792 | $8,073 | $7,225 | $7,481 | $8,082 | $7,219 |
| Incremental costs | -$7,519 | -$7,740 | -$15,033 | -$3,052 | -$9,842 | -$4,524 | -$11,153 | -$8,054 | -$9,006 |
|  |  |  |  |  |  |  |  |  |  |
| Costs by State and Territory government in usual care group | $1,522 | $1,523 | $1,523 | $1,522 | $1,522 | $1,522 | $1,523 | $1,522 | $1,522 |
| Costs by State and Territory government in optimal care group | $124 | $2,129 | $2,382 | $394 | $639 | $313 | $1,378 | $292 | $1,183 |
| Incremental costs | -$1,398 | $607 | $859 | -$1,128 | -$884 | -$1,209 | -$144 | -$1,230 | -$339 |
|  |  |  |  |  |  |  |  |  |  |
| Out-of-pocket costs in usual care group | $902 | $3,578 | $4,002 | $662 | $1,073 | $525 | $2,315 | $491 | $2,215 |
| Out-of-pocket costs in optimal care group | $88 | $504 | $570 | $51 | $115 | $30 | $308 | $25 | $292 |
| Incremental costs | -$814 | -$3,074 | -$3,432 | -$610 | -$958 | -$495 | -$2,008 | -$466 | -$1,923 |
|  |  |  |  |  |  |  |  |  |  |
| Total costs of compression therapy in usual care group^a^ | $3,431 | $3,434 | $3,435 | $3,431 | $3,433 | $3,433 | $3,433 | $3,432 | $3,433 |
| Total costs of compression therapy in optimal care group^b^ | $4,047 | $4,051 | $4,053 | $4,046 | $4,050 | $4,050 | $4,050 | $4,048 | $4,049 |
| Incremental costs | $615 | $617 | $618 | $615 | $617 | $617 | $617 | $616 | $617 |
|  |  |  |  |  |  |  |  |  |  |
| Total QALYs in usual care group | 3.04 | 3.04 | 3.04 | 3.04 | 3.04 | 3.04 | 3.04 | 3.04 | 3.04 |
| Total QALYs in optimal care group | 3.22 | 3.22 | 3.22 | 3.22 | 3.22 | 3.22 | 3.22 | 3.22 | 3.22 |
| Incremental QALYs | 0.18 | 0.18 | 0.18 | 0.18 | 0.18 | 0.18 | 0.18 | 0.18 | 0.18 |
| a Costs of compression therapy in usual care group were covered by out-of-pocket expenditure and government’s funding to health professionals.  b Costs of compression therapy in optimal care group were covered by Australian government | | | | | | | | | |

Table S10. Distribution of cost savings to the Australian government and State and Territory government

|  | NSW | VIC | QLD | SA | WA | NT | TAS | ACT |
| --- | --- | --- | --- | --- | --- | --- | --- | --- |
| %Cost savings to health system  (optimal care service delivery option 1) |  |  |  |  |  |  |  |  |
| Australian government | 84% | 80% | 91% | 58% | 87% | 73% | 88% | 85% |
| State and Territory government | 16% | 20% | 9% | 42% | 13% | 27% | 12% | 15% |
|  |  |  |  |  |  |  |  |  |
| %Cost savings to health system  (optimal care service delivery option 2) |  |  |  |  |  |  |  |  |
| Australian government | 85% | 81% | 90% | 69% | 87% | 77% | 88% | 86% |
| State and Territory government | 15% | 19% | 10% | 31% | 13% | 23% | 12% | 14% |

Table S11. CHEERS checklist

| **Section/item** | **Item No** | **Recommendation** | **Reported on page No/ line No** |
| --- | --- | --- | --- |
| **Title and abstract** | | | |
| Title | 1 | Identify the study as an economic evaluation or use more specific terms such as “cost-effectiveness analysis”, and describe the interventions compared. | page 1, line 1 to 2 |
| Abstract | 2 | Provide a structured summary of objectives, perspective, setting, methods (including study design and inputs), results (including base case and uncertainty analyses), and conclusions. | page 2, line 1 to 29 |
| **Introduction** | | | |
| Background and objectives | 3 | Provide an explicit statement of the broader context for the study. | page 3, line 1 to page 4, line 9 |
|  |  | Present the study question and its relevance for health policy or practice decisions. | page 4, line 10 to 15 |
| **Methods** | | | |
| Target population and subgroups | 4 | Describe characteristics of the base case population and subgroups analysed, including why they were chosen. | page 6, line 2 to 4 |
| Setting and location | 5 | State relevant aspects of the system(s) in which the decision(s) need(s) to be made. | page 5, line 18 to 22; |
| Study perspective | 6 | Describe the perspective of the study and relate this to the costs being evaluated. | page 8, line 9 |
| Comparators | 7 | Describe the interventions or strategies being compared and state why they were chosen. | page 4, line 19 to page 5, line 16 |
| Time horizon | 8 | State the time horizon(s) over which costs and consequences are being evaluated and say why appropriate. | page 6, line 1 to 2 |
| Discount rate | 9 | Report the choice of discount rate(s) used for costs and outcomes and say why appropriate. | page 6, line 21 to 22 |
| Choice of health outcomes | 10 | Describe what outcomes were used as the measure(s) of benefit in the evaluation and their relevance for the type of analysis performed. | Page 6, line 19 to 20 |
| Measurement of effectiveness | 11a | *Single study-based estimates:*Describe fully the design features of the single effectiveness study and why the single study was a sufficient source of clinical effectiveness data. |  |
|  | 11b | *Synthesis-based estimates*: Describe fully the methods used for identification of included studies and synthesis of clinical effectiveness data. | Page 6, line 25 to Page 8, line26 |
| Measurement and valuation of preference based outcomes | 12 | If applicable, describe the population and methods used to elicit preferences for outcomes. | not applicable |
| Estimating resources and costs | 13a | *Single study-based economic evaluation:*Describe approaches used to estimate resource use associated with the alternative interventions. Describe primary or secondary research methods for valuing each resource item in terms of its unit cost. Describe any adjustments made to approximate to opportunity costs. | Page 9, line 1 to line 9 |
|  | 13b | *Model-based economic evaluation:*Describe approaches and data sources used to estimate resource use associated with model health states. Describe primary or secondary research methods for valuing each resource item in terms of its unit cost. Describe any adjustments made to approximate to opportunity costs. |  |
| Currency, price date, and conversion | 14 | Report the dates of the estimated resource quantities and unit costs. Describe methods for adjusting estimated unit costs to the year of reported costs if necessary. Describe methods for converting costs into a common currency base and the exchange rate. | page 9, line 2 to 3 |
| Choice of model | 15 | Describe and give reasons for the specific type of decision-analytical model used. Providing a figure to show model structure is strongly recommended. | Page 5, line 18 to page 6, line 17 |
| Assumptions | 16 | Describe all structural or other assumptions underpinning the decision-analytical model. | page 5, line 4 to 7;  page 6, line 11 to 13;  page 8, line 18 to 20  page 9, line 12 to 15 |
| Analytical methods | 17 | Describe all analytical methods supporting the evaluation. This could include methods for dealing with skewed, missing, or censored data; extrapolation methods; methods for pooling data; approaches to validate or make adjustments (such as half cycle corrections) to a model; and methods for handling population heterogeneity and uncertainty. | page 10, line 11 to page 11, line 4 |
| **Results** | | | |
| Study parameters | 18 | Report the values, ranges, references, and, if used, probability distributions for all parameters. Report reasons or sources for distributions used to represent uncertainty where appropriate. Providing a table to show the input values is strongly recommended. | Table 1 |
| Incremental costs and outcomes | 19 | For each intervention, report mean values for the main categories of estimated costs and outcomes of interest, as well as mean differences between the comparator groups. If applicable, report incremental cost-effectiveness ratios. | Table 3 |
| Characterising uncertainty | 20a | *Single study-based economic evaluation:*Describe the effects of sampling uncertainty for the estimated incremental cost and incremental effectiveness parameters, together with the impact of methodological assumptions (such as discount rate, study perspective). |  |
|  | 20b | *Model-based economic evaluation:*Describe the effects on the results of uncertainty for all input parameters, and uncertainty related to the structure of the model and assumptions. | Page 14, line 1 to page 16, line 10 |
| Characterising heterogeneity | 21 | If applicable, report differences in costs, outcomes, or cost-effectiveness that can be explained by variations between subgroups of patients with different baseline characteristics or other observed variability in effects that are not reducible by more information. | not applicable |
| **Discussion** | | | |
| Study findings, limitations, generalisability, and current knowledge | 22 | Summarise key study findings and describe how they support the conclusions reached. Discuss limitations and the generalisability of the findings and how the findings fit with current knowledge. | page 16, line 14 to  page 20, line 4 |
| **Other** | | | |
| Source of funding | 23 | Describe how the study was funded and the role of the funder in the identification, design, conduct, and reporting of the analysis. Describe other non-monetary sources of support. | Page 21, line 7 to 11 |
| Conflicts of interest | 24 | Describe any potential for conflict of interest of study contributors in accordance with journal policy. In the absence of a journal policy, we recommend authors comply with International Committee of Medical Journal Editors recommendations. | Page 21, line 5 to 6 |


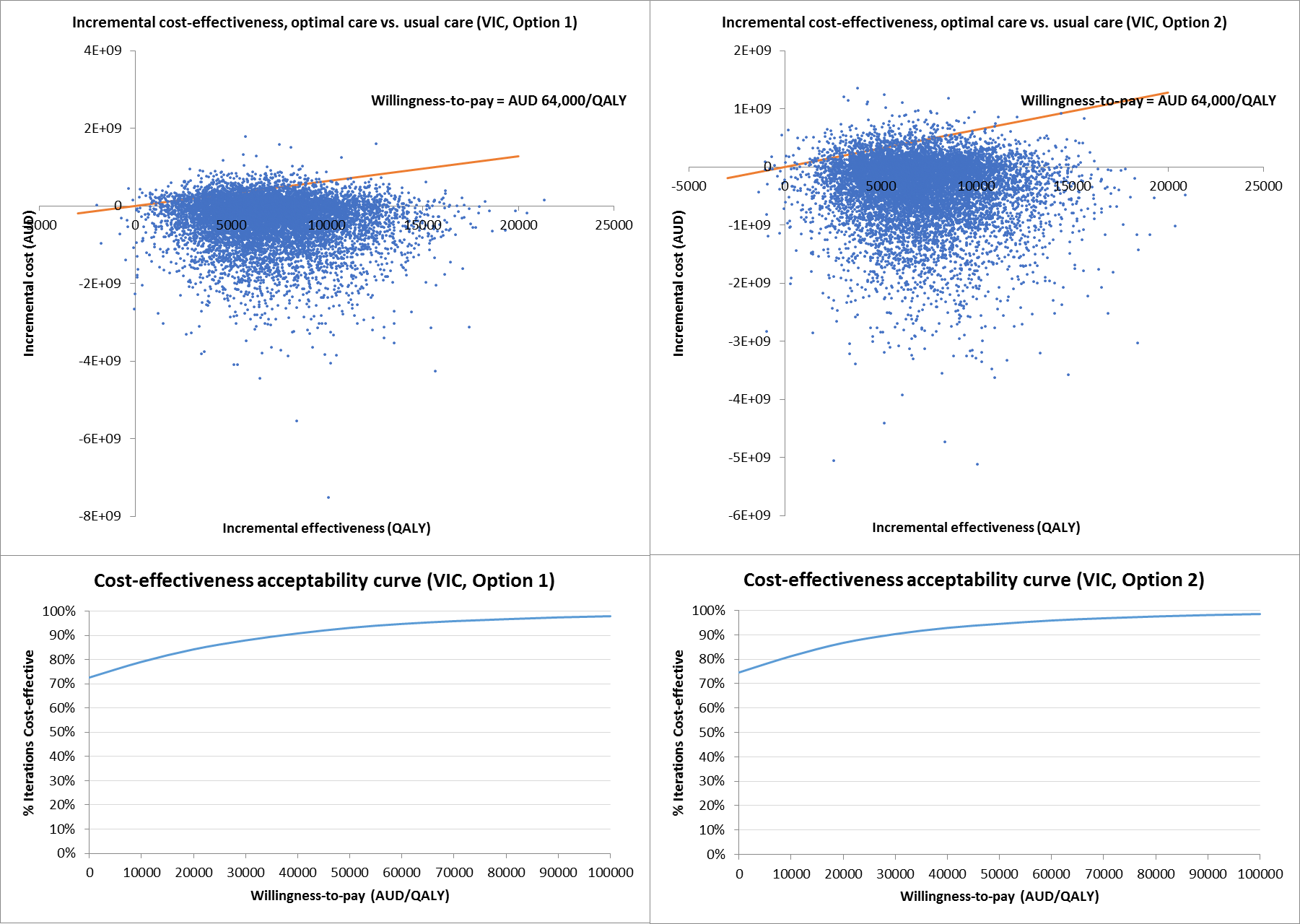


Figure S1. Probabilistic sensitivity analysis results for VIC


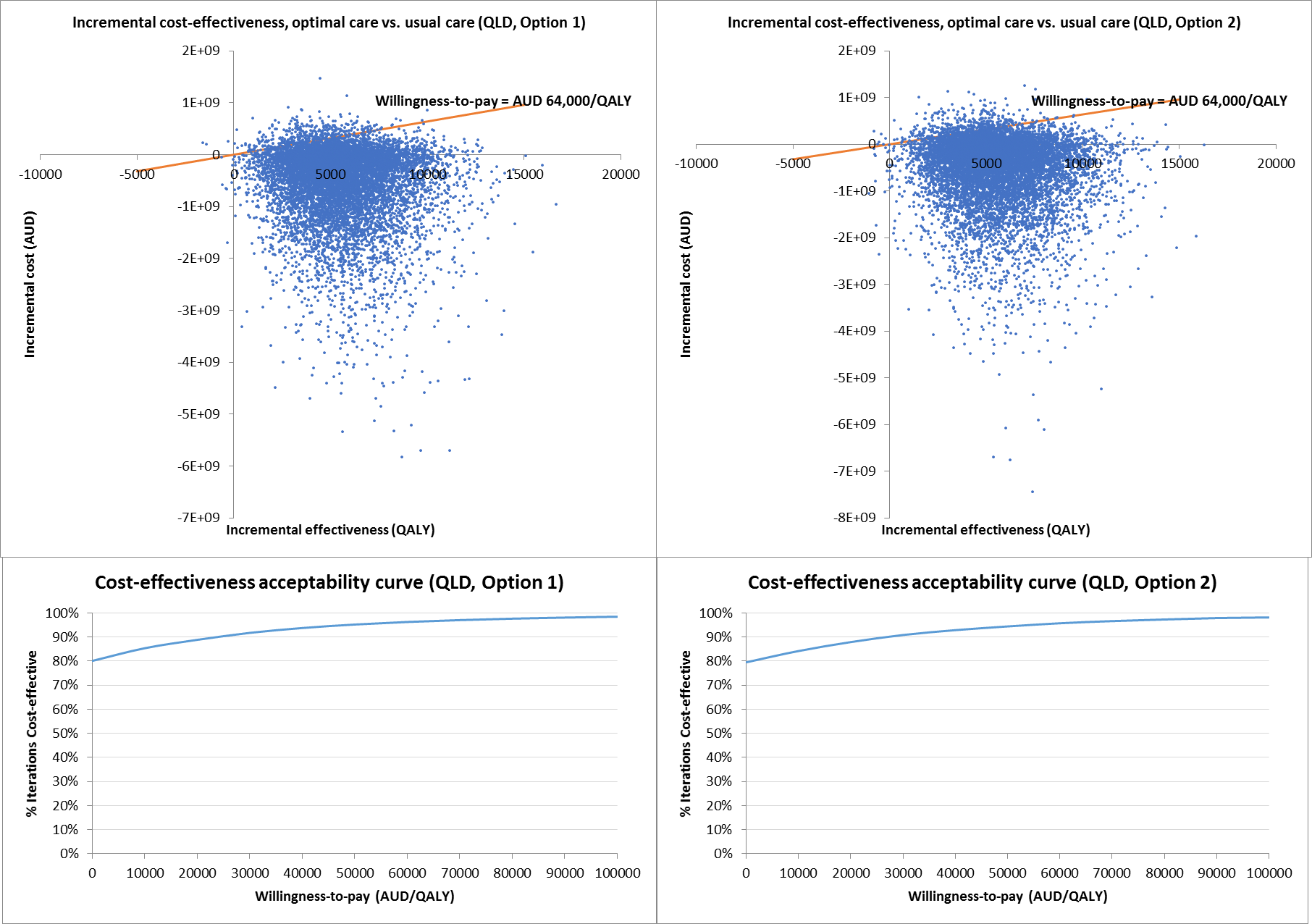


Figure S2. Probabilistic sensitivity analysis results for QLD


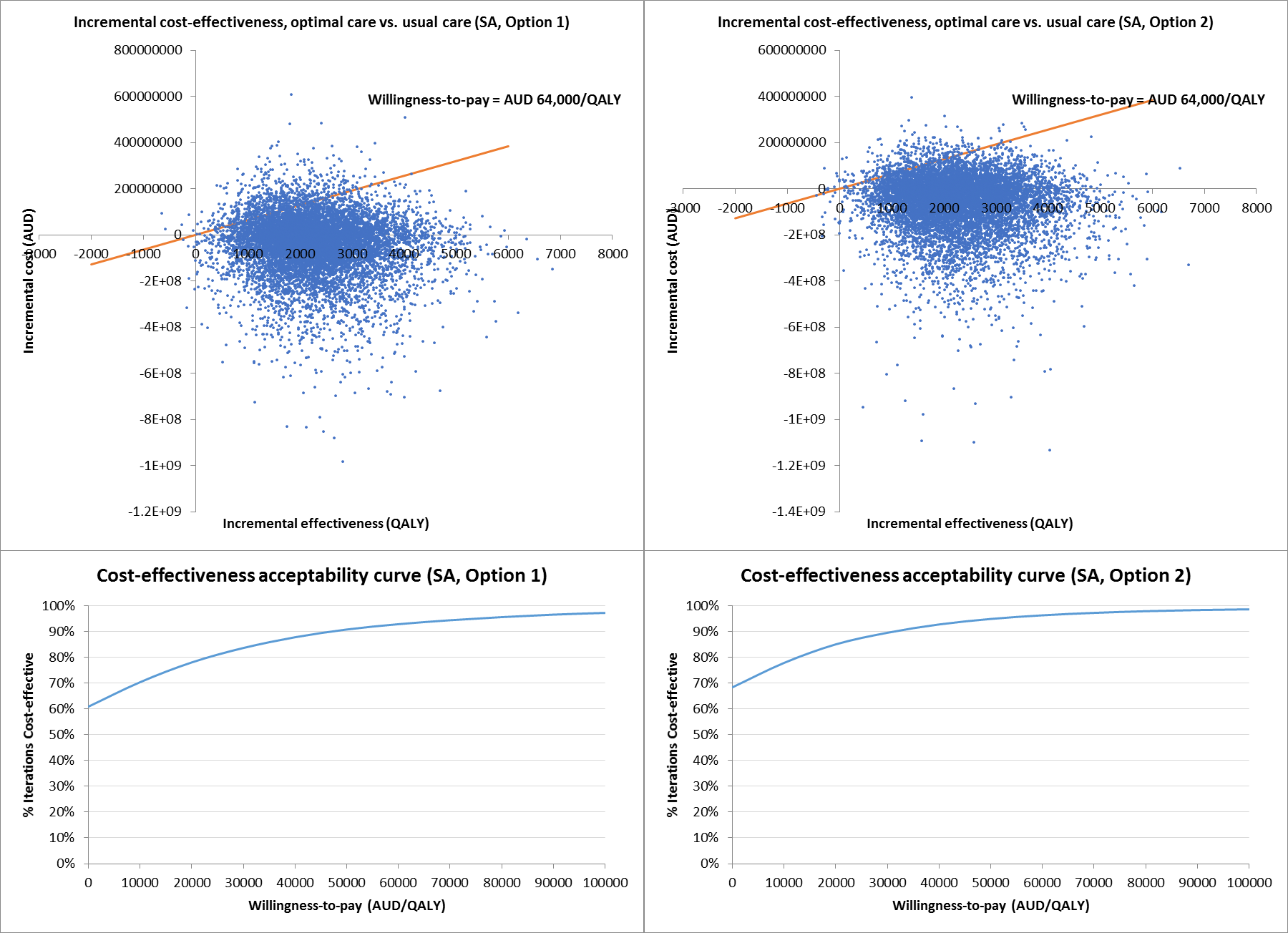


Figure S3. Probabilistic sensitivity analysis results for SA


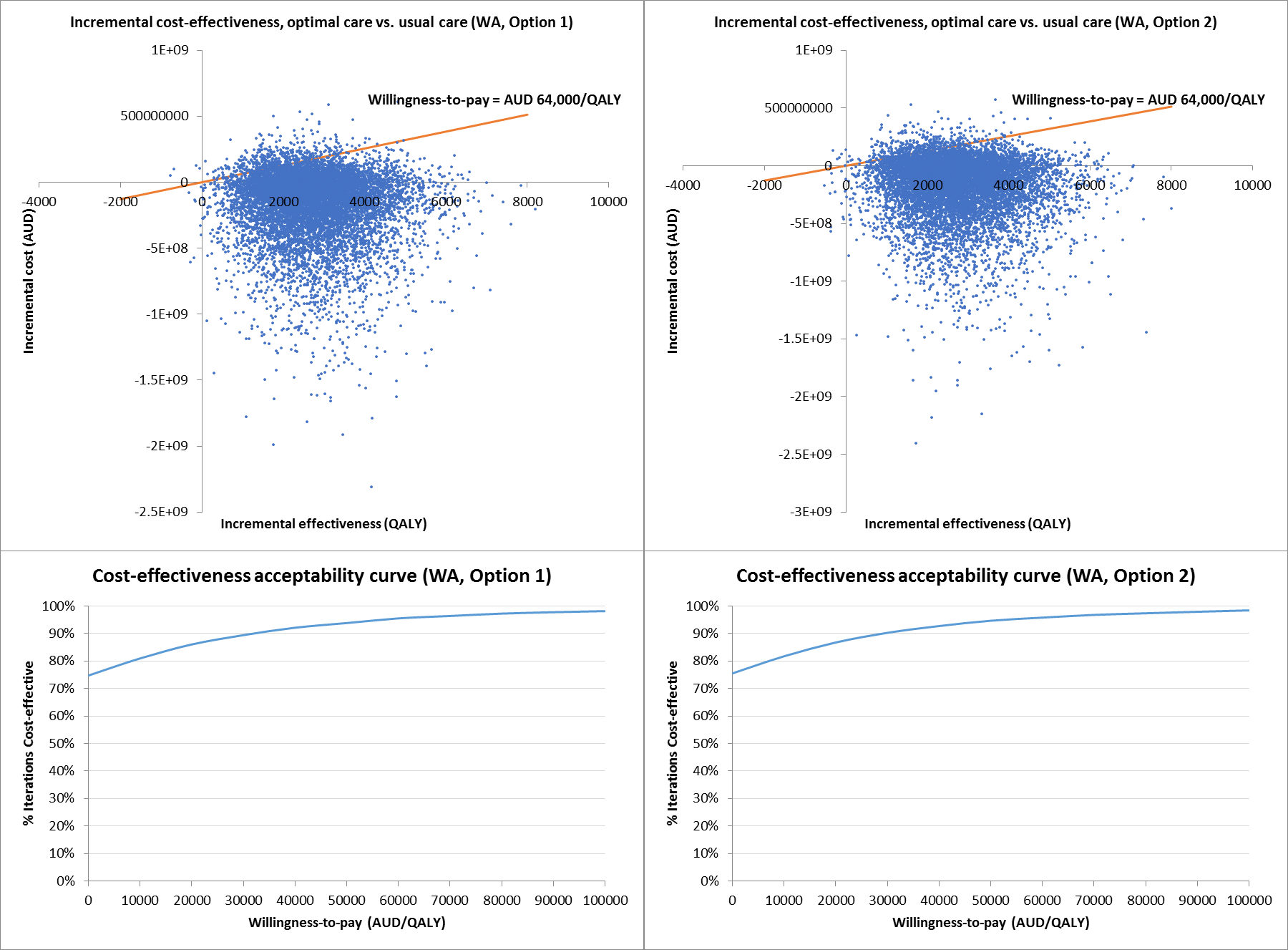


Figure S4. Probabilistic sensitivity analysis results for WA


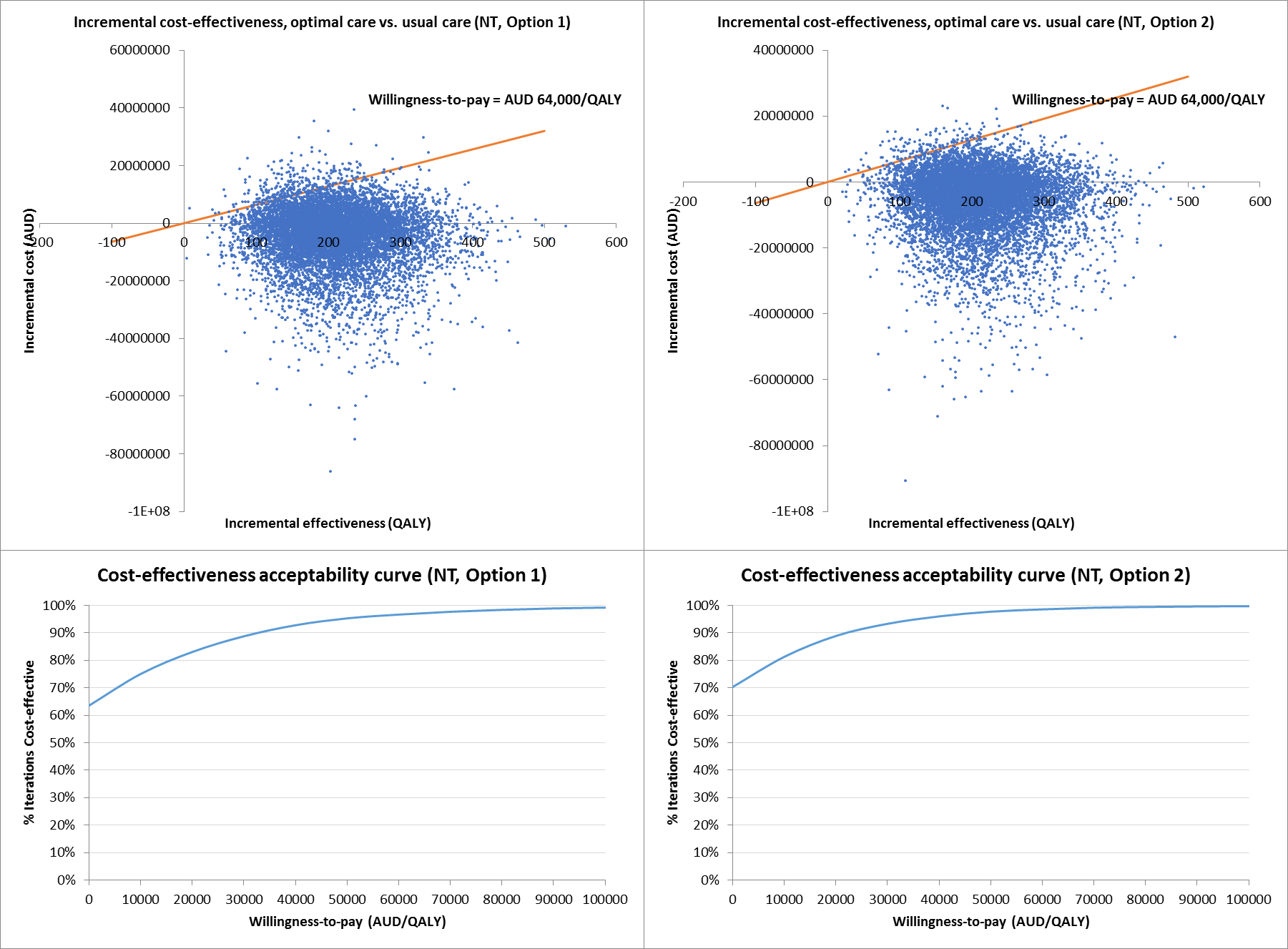


Figure S5. Probabilistic sensitivity analysis results for NT


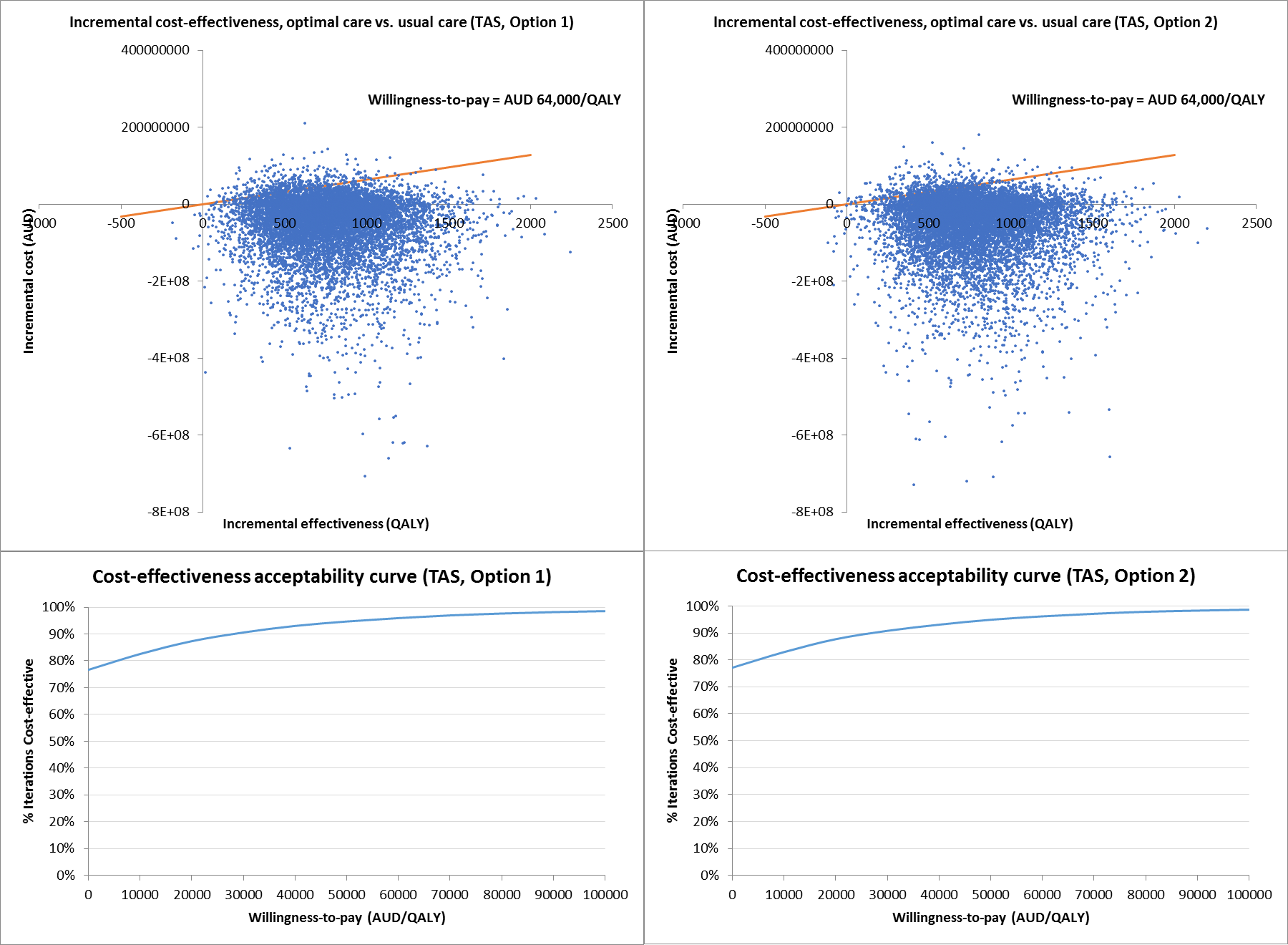


Figure S6. Probabilistic sensitivity analysis results for TAS


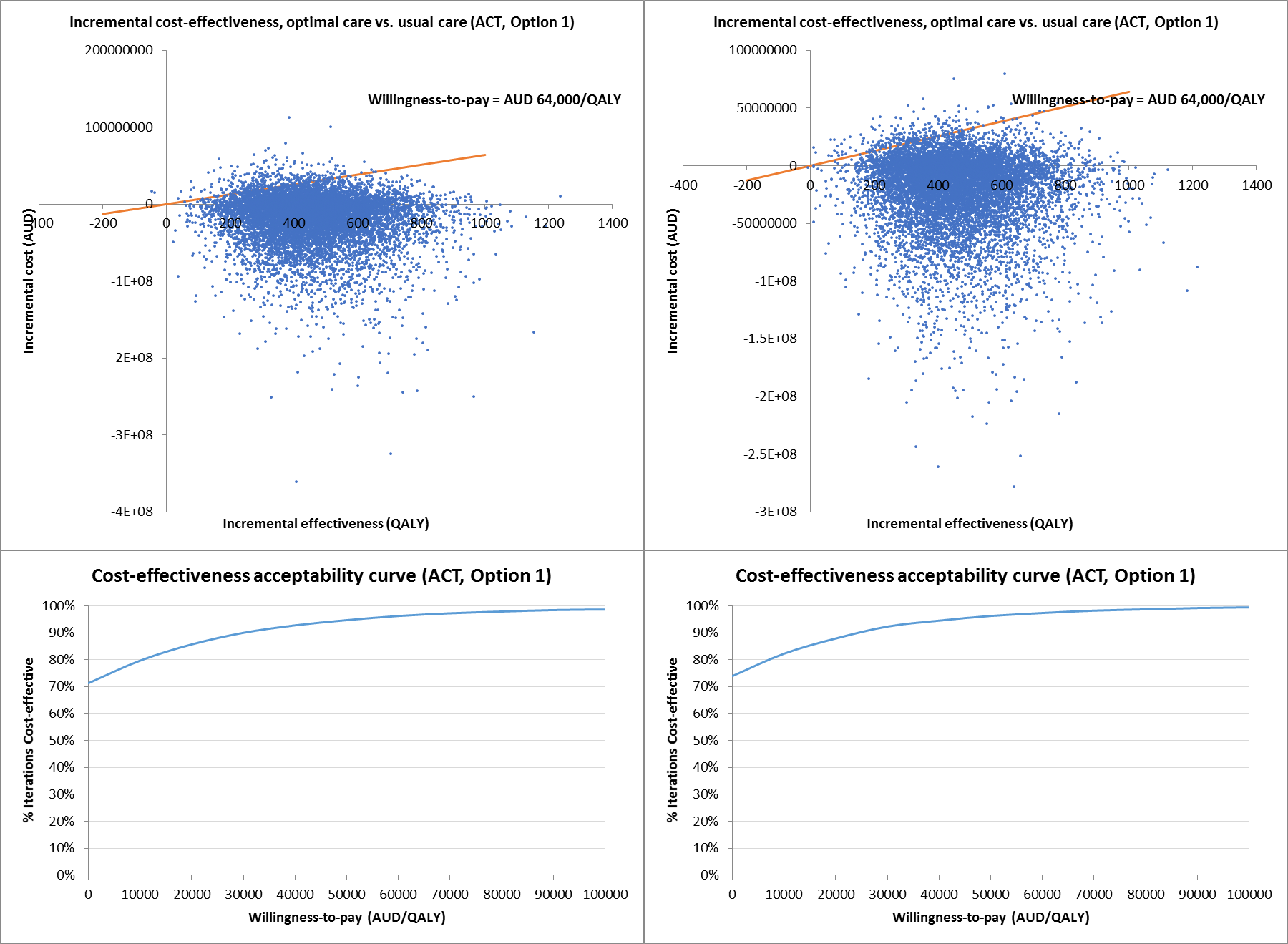


Figure S7. Probabilistic sensitivity analysis results for ACT

**References**

1. Australian Bureau of Statistics. Deaths, Year of occurrence, Age at death, Age-specific death rates, Sex, States, Territories and Australia 2017 [cited 2017 October 13]. Available from: http://stat.data.abs.gov.au/Index.aspx?DataSetCode=DEATHS_AGESPECIFIC_OCCURENCEYEAR.

2. Baker SR, Stacey MC. EPIDEMIOLOGY OF CHRONIC LEG ULCERS IN AUSTRALIA. Australian and New Zealand Journal of Surgery. 1994;64(4):258-61.

3. Australian Wound Management Association. KPMG Health Economics report: An economic evaluation of compression therapy for venous leg ulcers 2013. Available from: http://www.awma.com.au/publications/kpmg_report_brief_2013.pdf.

4. NSW Health. Increases to Salaries and Allowances – Public Health System and Crown Nurses' and Midwives' Awards 2015 [January 17, 2017]. Available from: http://www1.health.nsw.gov.au/PDS/pages/doc.aspx?dn=IB2015_034.

5. Fair Work Commission. Nurses and Midwives (Victorian Public Sector) (Single Interest Employers) Enterprise Agreement 2012-2016. 2012.

6. Queensland Health. Wage rates - Nursing Stream 2015 [January 17, 2017]. Available from: https://www.health.qld.gov.au/hrpolicies/wage_rates/nursing.

7. South Australian Industrial Relations Tribunals. Nurses (South Australian Local Government Sector) Award 2016 [January 17, 2017]. Available from: http://www.industrialcourt.sa.gov.au/index.cfm?objectid=7B94DD08-E7F2-2F96-3B05E8758A6CD135.

8. Western Australian Industrial Relations Commission. WA Health System – Australian Nursing Federation - Registered Nurses, Midwives, Enrolled (Mental Health) and Enrolled (Mothercraft) Nurses – Industrial Agreement 2016 2016 [January 17, 2017]. Available from: http://www.health.wa.gov.au/awardsandagreements/index.cfm#NR.

9. The Office of the Commissioner for Public Employment. Northern Territory Public Sector Nurses and Midwives' 2014-2017 Enterprise Agreement 2016 [Janaury 17, 2017]. Available from: https://ocpe.nt.gov.au/nt-public-sector-employment/enterprise-agreement-negotiations/rates-of-pay.

10. Department of Health and Human Services. Nurses and Midwives (Tasmanian State Service ) Agreement 2016 2016 [January 17, 2017]. Available from: http://www.dhhs.tas.gov.au/career/home/working_for_dhhs/salaries__and__benefits_150_overview/salary_rates/nurses_and_midwives.

11. ACT Health. ACT Public Service Nursing and Midwifery Enterprise Agreement 2013-2017 2014 [January 17, 2017]. Available from: http://www.health.act.gov.au/employment/enterprise-agreements/nursing-and-midwifery.

12. Kruger AJ, Raptis S, Fitridge RA. Management practices of Australian surgeons in the treatment of venous ulcers. ANZ journal of surgery. 2003;73(9):687-91.

13. Woodward M. Wound Management by Aged Care Specialists Primary Intention: The Australian Journal of Wound Management. 2002;10(2, May 2002):70-1, 3-6.

14. Independent Hospital Pricing Authority. National Hospital Cost Data Collection Australian Public Hospitals Cost Report 2014–2015, Round 19. 2017.
